# Technical considerations when designing a gene expression panel for renal transplant diagnosis

**DOI:** 10.1038/s41598-020-74794-3

**Published:** 2020-10-21

**Authors:** F. Toulza, K. Dominy, T. Cook, J. Galliford, J. Beadle, A. McLean, C. Roufosse

**Affiliations:** 1 Department of Immunology and Inflammation, Centre for Inflammatory Diseases, Faculty of Medicine, Imperial College, London, UK; 2 Molecular Pathology Laboratory, North West London Pathology, London, UK; 3 Imperial Kidney and Transplant Centre, London, UK

**Keywords:** Transplant immunology, Diagnostic markers, Predictive markers, Nephrology

## Abstract

Gene expression analysis is emerging as a new diagnostic tool in transplant pathology, in particular for the diagnosis of antibody-mediated rejection. Diagnostic gene expression panels are defined on the basis of their pathophysiological relevance, but also need to be tested for their robustness across different preservatives and analysis platforms. The aim of this study is the investigate the effect of tissue sampling and preservation on candidate genes included in a renal transplant diagnostic panel. Using the NanoString platform, we compared the expression of 219 genes in 51 samples, split for formalin-fixation and paraffin-embedding (FFPE) and RNAlater preservation (RNAlater). We found that overall, gene expression significantly correlated between FFPE and RNAlater samples. However, at the individual gene level, 46 of the 219 genes did not correlate across the 51 matched FFPE and RNAlater samples. Comparing gene expression results using NanoString and qRT-PCR for 18 genes in the same pool of RNA (RNAlater), we found a significant correlation in 17/18 genes. Our study indicates that, in samples from the same routine diagnostic renal transplant biopsy procedure split for FFPE and RNAlater, 21% of 219 genes of potential biological significance do not correlate in expression. Whether this is due to fixatives or tissue sampling, selection of gene panels for routine diagnosis should take this information into consideration.

## Introduction

Recent descriptions of the gene expression landscape in renal transplant biopsies have greatly advanced our understanding of immunological graft rejection. Gene expression analysis is now well on its way to becoming a diagnostic tool, as an element included in the Banff Classification for Renal Allograft Pathology^1^. Gene expression signatures of rejection were first discovered using microarray analysis^2^. However a number of research groups have now assessed gene expression signatures in transplant biopsies placed in an RNA preservative, using a variety of methods, not only microarray analysis^3–10^, but also quantitative real-time polymerase chain reaction (qRT-PCR)^11–13^ and RNA sequencing^9,14,15^. Further validation of gene signatures for diagnostic use has not proceeded however, partly because a renal transplant biopsy is an invasive procedure that yields a limited amount of tissue. Diagnostic evaluation of this precious sample requires that most of the sample be preserved in a fixative such as formalin for light microscopy, often also with separate samples for electron microscopy and/or immunofluorescence. The preservatives used for these investigations are not ideal for RNA preservation. There is reluctance from the renal community to either sacrifice any of these usual samples, or to take more tissue for RNA analysis, because this is associated with additional cost and increased potential risk and inconvenience for patients^12^. Therefore there has been considerable interest in a novel high throughput gene expression platform that works on formalin-fixed paraffin-embedded (FFPE) tissue, the NanoString nCounter Analysis System (NanoString Technologies, Seattle, WA, USA)^7,16–19^. Comparisons between results of gene expression analysis using Nanostring and qRT-PCR have been previously published, using optimally handled RNA from cell cultures and other organisms (sea urchins), or large tissue samples from humans (oral and lung tumours) mostly^7,16,17,20–23^. Two previous publications using renal transplant tissue^16,23^ analysed respectively 45 and 10 samples on small panels of 11 and 19 genes, comparing qPCR to Nanostring. We hypothesised that some genes may be more sensitive to variation introduced by sampling of different areas of the transplanted kidney, processing technique and/or gene expression detection method. The objectives of this study are to assess the effect on potential genes of interest in transplant diagnostics of: (1) sampling of different areas of the kidney for different sample preservatives (formalin-fixation with paraffin-embedding versus RNA preservative); and (2) RNA analysis technique (qRT-PCR or NanoString). We used genes from a list of potential genes of diagnostic interest selected from the literature, and samples from a real-life kidney transplant biopsy practice setting. We elected to analyse our samples with qRT-PCR and Nanostring, as both represent plausible techniques with relative ease of implementation in diagnostic laboratories; qRT-PCR is relatively cheap and easy and most diagnostic laboratories already have this platform and expertise in its use, whereas NanoString is of particular interest because of its optimisation for FFPE samples. This technical assessment is intended to inform future multi-centre diagnostic validation studies that will allow for the clinical adoption of molecular diagnostics in transplantation pathology.

## Results

### Comparison of gene expression levels in matched FFPE and RNAlater samples on the NanoString platform

We extracted RNA from 51 samples from the same biopsy procedure, split for FFPE and RNAlater. From FFPE samples, we obtained a median concentration of 111.39 ng/µl [36.5–271.5] with a median 280/260 nm of 2.05 [1.99–2.11]. From RNAlater samples, we obtained a median concentration of 125.78 ng/µl [53.3–366.7] with median a 280/260 nm of 1.97 [1.81–2.09]. RNA expression levels for 219 genes were measured using the NanoString platform on both samples. Data were normalized using the 11 housekeeping (HK) genes included in our panel (according to manufacturer recommendations) independently in the 51 samples in FFPE and in the 51 samples in RNAlater^20^.

**Figure 1:**
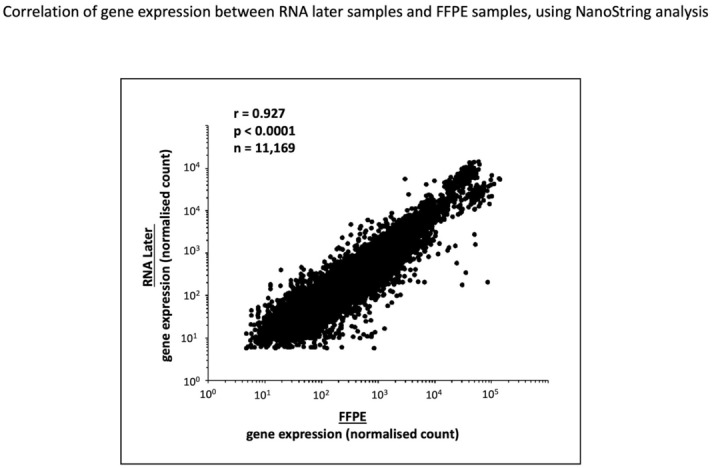
Correlation of gene expression between RNAlater samples and FFPE samples using the NanoString platform. The scatter plot shows expression results for up to 219 genes in 51 samples as quantified by Nanostring on the FFPE sample and corresponding RNAlater preserved biopsy. Statistic shows the Spearman rank correlation value.

**Figure 2:**
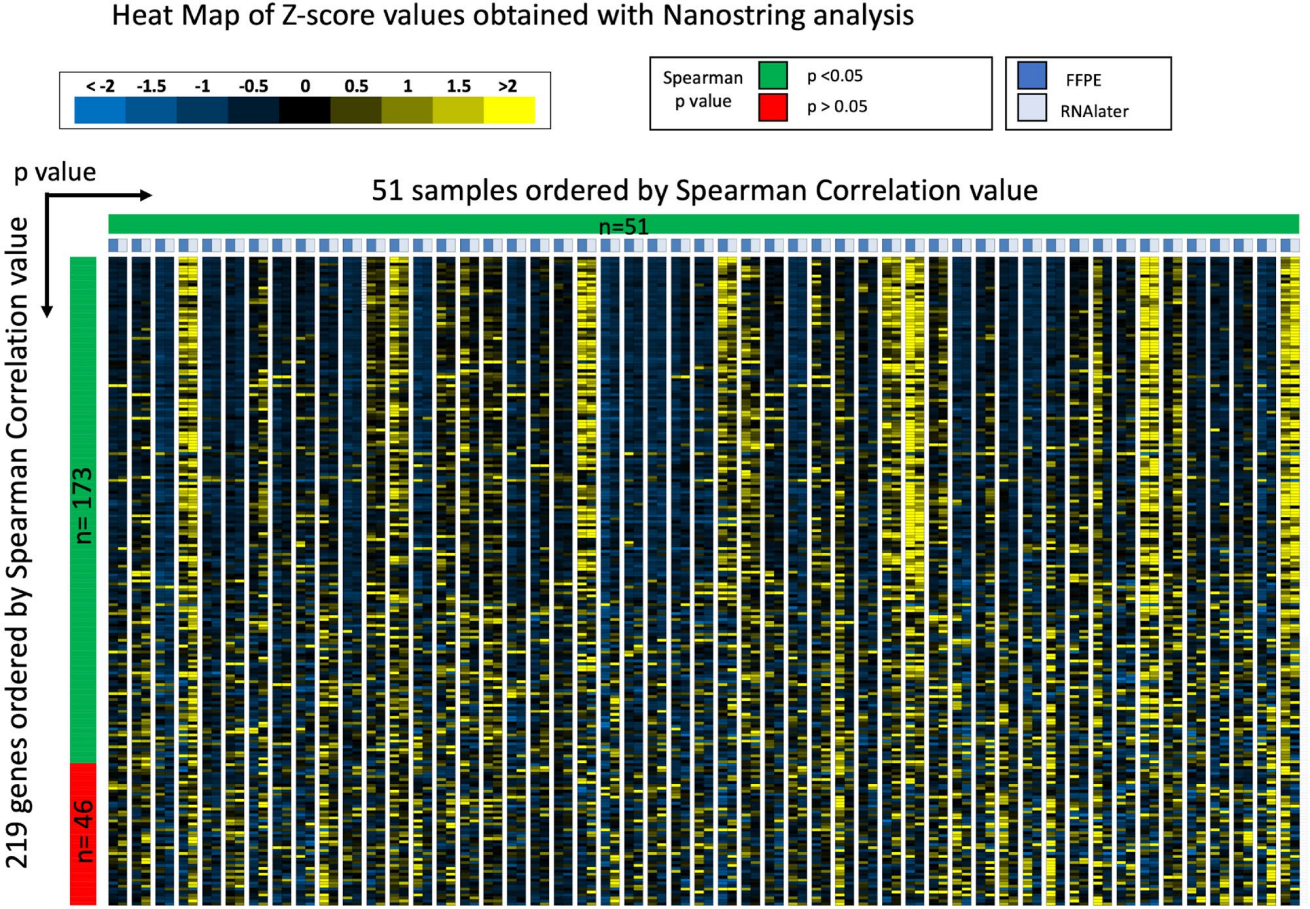
Representation by heat map of Z-score values obtained with NanoString analysis of FFPE and RNAlater matched samples. This graph represents the gene expression levels measured by Nanostring after Z-score transformation. Each row represents a gene, and each column represents a sample. Each column is double, with a dark blue box at the top of the column indicating Z-scores in the FFPE sample and a light blue box at the top of the column indicating Z-scores of the corresponding RNAlater sample. The heatmap colour range is from yellow for positive Z-scores values to blue for Z-score negative values. Samples are ordered horizontally from left to right according to their Spearman rank *p* value. Genes are ordered vertically from top to bottom by their Spearman rank *p* value. The results of the Spearman rank *p* value are also represented along the horizontal and vertical bars, where green represents *p* < 0.05 and red *p* > 0.05.

We first compared the levels of gene expression expressed as Z-scores, including all samples and all genes between FFPE and RNAlater using Spearman rank correlation analysis (Fig. 1). There was a strong correlation between gene expression in the 2 samples (correlation coefficient = 0.927; *p* < 0.001). We then assessed correlation for each individual sample and for each individual gene. The results are presented in a heat map graph in Fig. 2. This heatmap documents a wide range of expression values across the samples for most genes. Results of the Spearman rank analysis for each of the 51 individual samples between FFPE and RNAlater are expressed along the horizontal bar. We observed that in each of the 51 samples, there is a significant correlation between gene expression levels in the FFPE and RNAlater samples from the same kidney (r values range 0.814–0.979). This is represented by the fact that the horizontal bar is all green. Results of the Spearman rank analysis for each of the 219 individual genes between FFPE and RNAlater are expressed along the vertical bar. The r values for the genes ranged from − 0.11 to 0.886, and there was a significant correlation for 173/219 genes (79%). Of the 74 genes related to AMR, 24 (32%) did not show significant correlation (Supplemental Table 1). We hypothesised that genes with low expression could correlate less well or be more sensitive to degradation in FFPE but found no significant correlation between poor performance and the level of expression (Supplemental Table 2).

### Comparison of gene expression levels from the same pool of RNA analyzed with NanoString versus qRT-PCR

**Figure 3:**
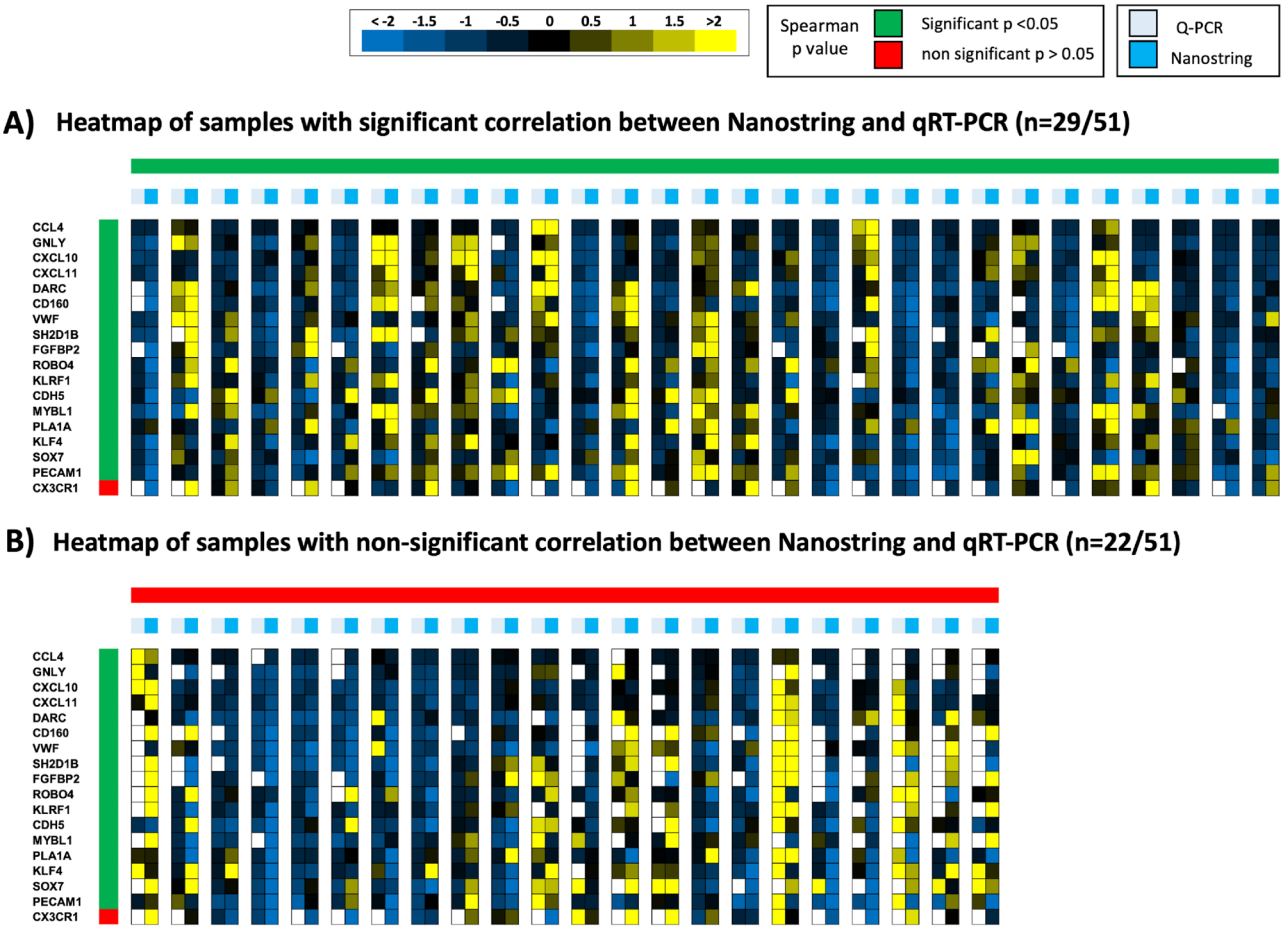
Representation by Heat Map of Z-score values from the same pool of RNA analyzed with NanoString versus qRT-PCR. Each row represents a gene, and each column represents a sample. Each column is double, with Z-scores in the NanoString analysis sample in light blue and Z-scores of the corresponding qRT-PCR dark blue. The heatmap color range is from yellow for positive Z-scores values to blue for Z-score negative values. Gene are ordered by their Spearman rank value (most significant on top; less significant at bottom). The white squares correspond to Non-Available data on qRT-PCR (NA) due to ct value higher than 34 or bad triplicate. Sample are grouped according to their Spearman rank *p* value: (**A**) samples with significant correlation between between NanoString and qRT-PCR for the expression of 18 genes, (**B**) samples with non-significant correlation between between NanoString and qRT-PCR for the expression of 18 genes.

For the 51 samples in RNAlater, we used part of the pool of RNA for NanoString analysis, and part for qRT-PCR analysis. This was only performed for 18 genes, as described in Methods. Only RNAlater samples were tested by qRT-PCR, as the RNA quality from FFPE was not of sufficient quality. We first compared the levels of gene expression expressed as Z-scores, including all samples and all genes between qRT-PCR and NanoString using Spearman rank correlation analysis (Supplemental Fig. 1) and found a significant correlation (r = 0.588 *p* < 0.001). We then analysed individual samples and individual genes (Fig. 3). The horizontal axis shows results of Spearman rank correlation analysis for Z-scores using NanoString or qRT-PCR for each of the 51 samples. We found a significant correlation between the NanoString and qRT-PCR measurements for only 29/51 samples (56.8%). The vertical axis in Figs. 3A,B illustrates Spearman rank correlation analysis of Z-scores for each of the 18 genes, comparing expression levels obtained with NanoString and qRT-PCR. We found a significant correlation for 17/18 genes (94%), with *CX3CR1* being the one exception. *CX3CR1* levels correlated significantly when performing NanoString analysis on matched FFPE and RNAlater samples in Fig. 2 (detailed data also shown in Supplemental Fig. 1). We believe the lack of correlation here is related to a qRT-PCR primer problem, as this set of primers failed to work in 50% of samples. Notably, 2 AMR genes (*CDH5* and *SOX7*) that correlated well between qRT-PCR and NanoString on the same sample did not correlate with each other when comparing a different pool of RNA (FFPE or RNAlater, from the same biopsy procedure) using NanoString. This suggests a susceptibility of these genes to either sampling of different areas of the kidney, and/or to FFPE preservation.

We noted that for qRT-PCR, there were many samples with missing values, related to poor triplicates (Ct Standard deviation > 1) or ct values of > 34, and missing values were enriched for some genes. Some samples were also enriched for missing values (most samples had 0–4 qRT-PCR values missing, but 12/51 had 5 or more values missing) despite optimisation of primers. We checked if genes with a high number of missing values were associated with a low count in Nanostring in corresponding samples, but there was no correlation (data not shown). This likely explains to a large extent the presence of so many samples with poor correlation between qRT-PCR and Nanostring.

### Ability to distinguish rejection (R) from Non-rejection (NR)

**Figure 4:**
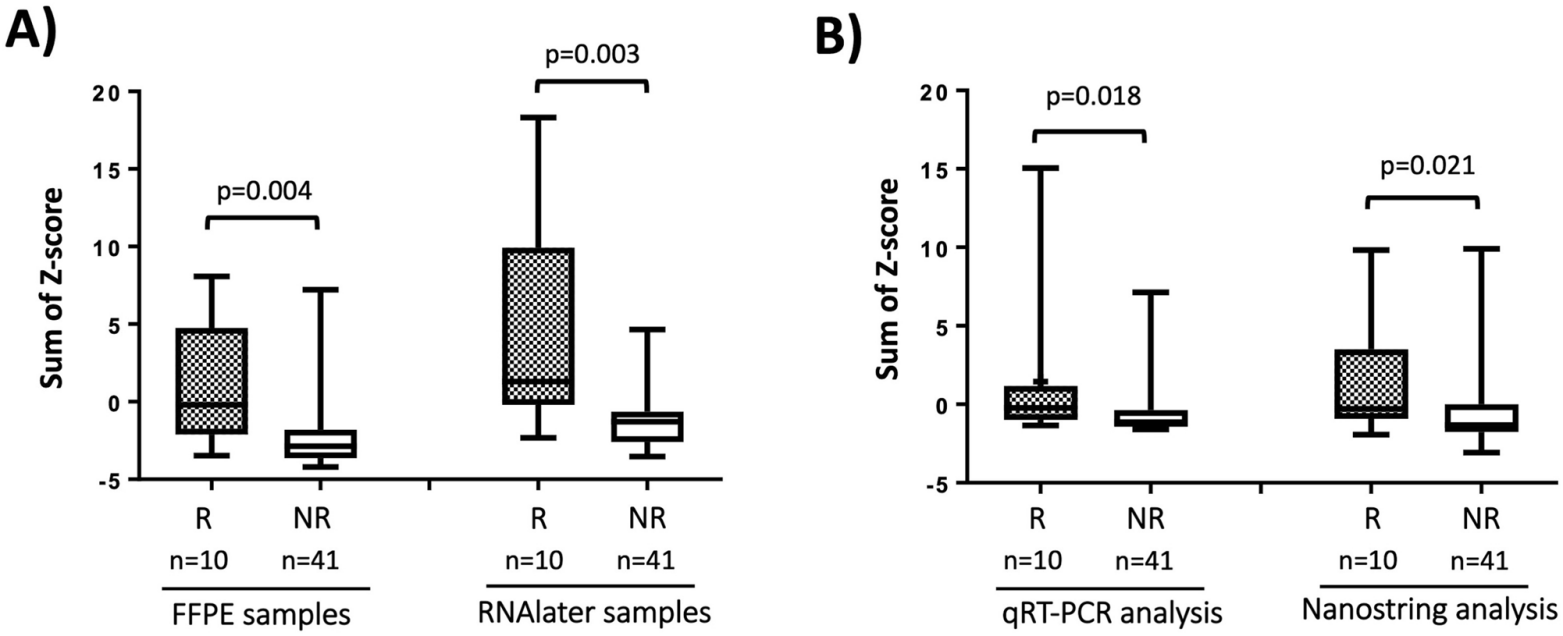
(**A**) Ability of NanoString analysis of FFPE and RNAlater samples to distinguish Rejection (R) from Non-rejection (NR); (**B**) ability of NanoString and qRT-PCR to differentiate R from NR in RNAlater preserved biopsies. Each box plot represents the distribution of the sum of Z-score for genes increased in rejection. Grey boxes represent 10 rejection samples (R) and white boxes represent 41 non-rejections samples (NR). (**A**) sum of z-score of 6 genes (*CCL4*, *CXCL9*, *CXCL10*, *CXCL11*, *IDO1* and *PLA1A*) comparing FFPE and RNAlater samples from the same biopsy; (**B**) sum of z-scores of 4 genes (*CCL4*, *CXCL10*, *CXCL11* and *PLA1A*) comparing qRT-PCR and NanoString on RNA from the same RNAlater sample. Significance determined using Mann Whitney test.

Of the 51 samples analysed, 10 showed histological rejection (R). The 41 samples not classified as rejection (NR) showed a variety of pathologies: acute tubular injury or fibrosis with no significant inflammation (n = 27) glomerulonephritis (n = 9), pyelonephritis (n = 4) or thrombosis (n = 1). We tested for genes canonical for rejection *CCL4*, *CXCL9*, *CXCL10*, *CXCL11*, *IDO1* and *PLA1A*^3,24^. We were not able to assess the AMR gene signature because only 2 samples showed signs of AMR. As previously described^11^, we calculated the sum of Z-scores for these 6 genes and compared R and NR samples. The results presented in Fig. 4A show that the sum of Z-scores using Nanostring analysis is significantly higher in R compared to NR using both FFPE (*p* = 0.004) and RNAlater samples (*p* = 0.003). We measured the sum of Z-scores for 4 genes (*CCL4*, *CXCL10*, *CXCL11* and *PLA1A*) using both qRT-PCR and NanoString. The results presented in Fig. 4B show that the sum of Z-scores is significantly higher in R compared to NR using both techniques (*p* = 0.018 and 0.021 respectively). There was an outlier in the non-rejection category which was a case of pyelonephritis which might also have comprised an un-recognised alloimmune element.

## Discussion

Our study indicates that, in samples from the same routine diagnostic renal transplant biopsy procedure split for FFPE and RNAlater, only a subset of all genes of potential biological significance (173/219, 79%) show significant correlation in expression between the 2 samples. Whether this is due to fixatives or tissue sampling, selection of gene panels for routine diagnosis should take this information into consideration. When comparing gene expression results using different platforms on the same pool of RNA from a sample in RNAlater (the ideal way to handle tissue for RNA preservation), we found a significant correlation in 17/18 genes, with the only discrepant gene CX3CR1. For this gene, we suspect a problem with the primers, as 50% of experiments failed.

Two previous studies have carried out similar investigations with fewer samples (n = 10–45), and fewer genes (n = 11–19)^7,16,23^. Adam et al. analysed 34 genes in 56 FFPE samples using Nanostring, with corresponding qRT-PCR data for 11 genes in 45 RNAlater matched samples^16,23^. This study primarily investigated variation between platforms, and because of its design, could not investigate the effect of preservative (FFPE versus RNAlater) on gene expression. They found a moderate correlation between Nanostring and qRT-PCR comparing 11 genes in 45 samples (r = 0.487, *p* < 0.001), but differences may have been due to platform, tissue sampling or tissue preservation. Greater correlation between Nanostring results and histological features of rejection was found, which, in combination with variable correlation coefficients from sample to sample (0.848–0.999), suggests that sampling of different areas of the kidney for FFPE and RNAlater played a role in the discrepant results. Sigdel et al. compared expression of 19 genes in 10 samples, using both qRTPCR and Nanostring, in matched cores in FFPE and RNAlater^7^. When comparing gene expression levels between samples placed in different preservatives (FFPE/RNAlater) on the Nanostring platform, Sigdel et al. found a mean r value of 0.82, with a range of 0.45–0.96 depending on the individual sample. These findings are similar to ours; we found a correlation overall across all genes and samples of r = 0.93, with a range of r values depending on the individual sample of 0.81–0.99. Sigdel et al. only investigated 19 genes of interest and did not provide individual r values for each gene. Our data adds to this field, by demonstrating on a wide range of genes (n = 219) that not all biologically relevant genes considered for diagnostic panels show a similarly robust expression across samples. We found that for 46/219 genes (including 24/74 genes described as AMR-associated), there was poor correlation between the sample in RNAlater and the FFPE sample. This was not related to low gene expression. Possible explanations are susceptibility of some individual transcripts to specimen handling (RNAlater versus FFPE), or a sampling issue, as the 2 pools of RNA for FFPE and RNAlater analysis came from 2 different parts of the renal biopsy sample. An association has been described between the expression level of glomerular podocyte-specific transcript NPHS2 (podocin) and the proportion of cortex in the biopsy^25^. In our data, the expression of NPHS2 significantly correlated between the FFPE and RL samples (*p* = 0.038). Although not conclusive, this finding suggests a rough correlation between the 2 samples for cortical sampling across the dataset (supplemental Table 1). If we can’t absolutely exclude discrepancy due to sampling of a different zone of biopsy, we found that only 46 genes did do not correlate, suggesting that 173 genes are not impacted by the location of the biopsy sample.

Either way, our data suggest that some genes may be better candidates for reference/consensus diagnostic panels than others. We supply a list (Supplemental Table 1) of potentially less reliable AMR genes, as they do not correlate between the 2 preservation methods tested in this study.

We also add further data to the evidence provided by Sigdel et al. and Adam et al., in support of the finding that choice of platform is not an issue in routine diagnostic transplant biopsies. Indeed we found a robust association between qRT-PCR and Nanostring results on the same pool of RNA, including between some genes that did not correlate well when comparing Nanostring analysis from different samples of the same kidney (in this case, *CDH5* and *SOX7).*

The selection of appropriate housekeeping (HK) genes for normalisation is an important step in the analysis. We observed that the half of the HK genes we tested did not correlate well between FFPE and RNAlater preserved samples (Supplemental Table 3). Others have noted, using RNAseq, that the expression of some HK genes can vary depending on the renal pathology present in the biopsy^26^. HK genes for diagnostic use will need to be optimised according to their susceptibility to variation related not only of diagnosis but also to tissue sampling and preservation. We contribute to this optimisation, by providing information on the 6 HK genes that, in our hands, were not impacted by preservation method (SDHA, GUSB, ACTB, LDHA, DDX50 and HPRT1). Further investigation by other groups will be needed to corroborate our findings. We suggest that future reports must specifically include data on expression levels of HK genes. We don’t know why we find a difference in expression in HK genes, but suspect the FFPE preservation method could impact the stability of some HK genes, as was previously observed in a study of sarcoma FFPE samples^27^.

The transplant community is keen to adapt the new molecular understanding of rejection for diagnostic use. However, both consensus validated diagnostic gene lists and tissue sampling pathways need to be determined. Reassuringly, in alignment with previous studies, we were also able to detect a significant difference in rejection-related gene expression between samples with and without rejection, using 2 techniques and 2 fixatives. Regarding tissue sampling, on the one hand, a portion of the scanty tissue obtained during the biopsy procedure could be placed in an RNA preservative, an approach renal biopsy-takers are already familiar with as most renal samples are already divided to yield small portions of cortex for immunofluorescence and electron microscopy. On the other hand, with the advent of techniques such as NanoString that are applicable to FFPE tissue, left over material from the FFPE block after routine diagnosis could be used for molecular analysis. Regarding platform, there is current enthousiasm in the transplant field for adoption of NanoString-based analysis, in particular because of its suitability to FFPE tissue. However, this platform requires additional equipment and reagents, whereas qRT-PCR can be easily performed at low cost in any molecular laboratory. In our qRT-PCR analysis of samples in RNAlater, there were many missing values. This is an important drawback given that gene expression analysis for the diagnosis of rejection requires not a single transcript but a combined score from a panel of genes. Missing values in qRT-PCR are likely to be a problem in a routine diagnostic setting. In order to make use of qRT-PCR with its advantages (cheap, flexible and fast method that can be performed on a single sample at a time), we will need to choose genes with a robust assay and/or design a score that allows for an occasional missing value. An optimal clinical diagnostic test would allow for local choices in both tissue preservation and RNA analysis platform, with similar results obtained irrespective of these.

A limitation of our study is that, due to the random nature of samples available in RNAlater, we only included 2 cases of AMR, so we could not explore differences in expression of AMR-related genes in a useful set of biopsies, even though such a panel is the most interesting for diagnostic use at this stage. A larger study with a high number of AMR samples will be required.

We conclude that whatever the sample preservative or RNA analysis technique used, it is possible to detect a significant difference between rejection and non-rejection samples using appropriate gene expression panels. However, we also find that tissue preservative and/or sampling has an influence on the expression of a subset of genes from the Banff Molecular Group panel, including some housekeeping genes and some genes associated with antibody-mediated rejection. The realities of clinical practice are such that as new diagnostic techniques are introduced, local expertise, available platforms and personal preferences may end up dictating the sample preservation and gene expression analysis platform used. In the field of transplant rejection diagnosis, where a panel of genes is needed rather than a single one, local validation of the diagnostic test will be facilitated by data such as that presented here, that document which genes are prone to variations across different platforms, different sample preservation techniques and/or sampling of different areas of the kidney. Important next steps will include evaluation of inter-laboratory reproducibility, including the use of synthetic standards.

## Methods

### Sample collection

Renal transplant tissue was obtained from the Imperial College Healthcare NHS Trust Tissue Bank, which has ethics approval to both collect human tissue and release material to researchers (MREC 17/WA/0161). All experiments were performed according the NHS Trust Tissue Bank regulation. Fifty-one routine diagnostic renal transplant biopsy cores were obtained under ultrasound guidance with an 18-gauge spring-loaded needle. Most of the sample was placed as per diagnostic protocol in formalin for between 3 and 20 h. Allocation of cores for RNAlater (Life Technologies, Paisley, UK) was variable depending on each individual case; either one full core or a portion of 1 core (divided transversally) was selected. This was performed at bedside by the attending nephrologist, without the aid of a dissecting microscope. Material left over after diagnosis was complete was used for RNA extraction. Banff lesion scores and diagnostic categories were recorded by a pathologist (C.R.).

### RNA extraction

Formalin-Fixed Paraffin-Embedded (FFPE) blocks: Consecutive 20-μm curls (between 4 and 6) were obtained. Microtome blades were replaced, and equipment was cleaned with RNaseZap (Life Technologies, Paisley, UK) between each block. Sections were immediately transferred to RNase-free 1.5-ml microcentrifuge tubes and placed on ice. RNA was extracted using the RNeasy FFPE kit (Qiagen, Hilden, Germany) and deparaffinization solution (Qiagen, Germany) according to the manufacturer’s protocol. RNA concentration and purity were quantified with a NanoDrop 2000c Spectrophotometer (LabTech, East Sussex, UK).

RNALater samples: RNA was extracted from the whole tissue using Trizol (Life Technologies, Paisley, UK), purified using RNA Mini Kit (Qiagen, Germany) according to the manufacturer’s protocol, and quantified with a NanoDrop 2000c Spectrophotometer (LabTech, East Sussex, UK).

### Quantitative real-time PCR (qRT-PCR)

Up to 1 μg RNA was converted to complementary DNA using an iScript select kit (Bio-Rad, Hemel Hempstead, UK) with random hexamer priming. qRT-PCR was carried out using an Applied Biosystems 7500 real-time qPCR machine. Ten nanograms complementary DNA was used in SYBR green assay (Agilent Technologies) with gene-specific primers spanning an intron for 18 genes (Supplemental Table 4)^24,28^. This selection of 18 genes represented genes in the Banff Molecular Working Group reference panel that were high-lighted in several publications as related to rejection, and in particular antibody-mediated rejection. Only 18 genes could be tested due to limited material available, so we restricted our choice to genes increased in antibody-mediated rejection, as these are the most likely to be clinically useful in the near future. Primers were designed to map to the same region of mRNA as the NanoString probe. Reactions were performed in triplicate at 95 °C for 10 min, followed by 40 cycles of 95 °C for 15 s, and 60 °C for 1 min. A threshold cycle (CT) was recorded in the exponential phase of amplification, and melt curves were created to confirm primer specificity (15 s 95 °C, 1 min 60 °C increasing at 0.05 °C/second to 95 °C for 15 s). Gene expression levels were normalised to housekeeping gene HPRT.

### NanoString gene expression analysis

We selected 219 genes from the Banff Molecular Working Group reference panel^28,29^. This selection of 219 genes represented those genes in the Banff list that were high-lighted in several publications as related to rejection. A custom nCounter XT CodeSet (NanoString Technologies, Seattle, WA) (Supplemental Table 5) was used to analyse gene expression in both FFPE and RNAlater samples. Quality control and normalization of raw gene expression counts were performed with nSolver Analysis Software Version 4.0 (NanoString Technologies). Default parameters for quality control flagging were used for imaging (field of view registration > 75%), binding density (0.05–2.25), positive control linearity (R2 value > 0.95), and positive control limit of detection (0.5 fM positive control > 2 SDs above the mean of the negative controls) as previously described^16^. Background subtraction was performed for each sample by subtracting the mean of the negative controls from all data points.

### Data analysis

GraphPad Prism 7.02 (GraphPad, La Jolla, CA) and IBM SPSS 10 (IBM Corp, Chicago, IL) were used to analyse results. To compare gene expression values obtained with 2 different techniques with differing ranges of values, we used Spearman rank, with significance set at *p* < 0.05. To compare gene expression levels between genes with widely differing levels of expression, we performed for each gene a Z-score transformation across all samples tested.

Gene expression levels between rejection and non-rejection groups were compared using a 2-tailed Mann–Whitney test.

Analysis of relative gene expression data using qRT-PCR and the 2(− Delta Delta C(T))^30^ was performed in Excel. Expression data for NanoString and qRT-PCR were normalized onto the same scale for each gene by calculating a Z-score (Z = (X–mean)/Standard deviation), with X = gene expression provided by nSolver analysis software or relative gene expression from qRT-PCR.

## Supplementary information

Supplementary Figure.

Supplementary Table 1.

Supplementary Table 2.

Supplementary Table 3.

Supplementary Table 4.

Supplementary Table 5.

## Abbreviations

AMR: Antibody-mediated rejection
DSA: Donor-specific antibody
FFPE: Formalin-fixation and paraffin-embedding
HK: Housekeeping
NR: Non-rejection
qRTPCR: Quantitative real-time polymerase chain reaction
R: Rejection

## References

[1] Haas M (2014). Banff 2013 meeting report: inclusion of c4d-negative antibody-mediated rejection and antibody-associated arterial lesions. Am. J. Transplant.

[2] Halloran PF, Einecke G (2006). Microarrays and transcriptome analysis in renal transplantation. Nat. Clin. Pract. Nephrol..

[3] Halloran PF, Famulski K, Reeve J (2015). The molecular phenotypes of rejection in kidney transplant biopsies. Curr. Opin. Organ. Transplant..

[4] Suviolahti E (2015). Genes associated with antibody-dependent cell activation are overexpressed in renal biopsies from patients with antibody-mediated rejection. Transpl. Immunol..

[5] Sellares J (2013). Molecular diagnosis of antibody-mediated rejection in human kidney transplants. Am. J. Transplant..

[6] Hayde N (2014). Increased intragraft rejection-associated gene transcripts in patients with donor-specific antibodies and normal biopsies. Kidney Int..

[7] Sigdel TK (2018). Targeted transcriptional profiling of kidney transplant biopsies. Kidney Int. Rep..

[8] Modena BD (2016). Gene expression in biopsies of acute rejection and interstitial fibrosis/tubular atrophy reveals highly shared mechanisms that correlate with worse long-term outcomes. Am. J. Transplant..

[9] Thareja G (2018). Single nucleotide variant counts computed from RNA sequencing and cellular traffic into human kidney allografts. Am. J. Transplant..

[10] Sarwal M (2003). Molecular heterogeneity in acute renal allograft rejection identified by DNA microarray profiling. N. Engl. J. Med..

[11] Dominy KM (2015). Use of quantitative real time polymerase chain reaction to assess gene transcripts associated with antibody-mediated rejection of kidney transplants. Transplantation.

[12] Allanach K (2008). Comparing microarray versus RT-PCR assessment of renal allograft biopsies: similar performance despite different dynamic ranges. Am. J. Transplant..

[13] Sigdel TK (2015). A 15llografts. PLoS ONE.

[14] Cippà PE (2018). Transcriptional trajectories of human kidney injury progression. JCI Insight.

[15] Mueller FB (2019). Landscape of innate immune system transcriptome and acute T cell-mediated rejection of human kidney allografts. JCI Insight.

[16] Adam B (2016). Multiplexed color-coded probe-based gene expression assessment for clinical molecular diagnostics in formalin-fixed paraffin-embedded human renal allograft tissue. Clin. Transplant..

[17] Reis PP (2011). mRNA transcript quantification in archival samples using multiplexed, color-coded probes. BMC Biotechnol..

[18] Dominy KM (2019). Molecular assessment of C4d-positive renal transplant biopsies without evidence of rejection. Kidney Int. Rep..

[19] Oghumu S (2016). Differential gene expression pattern in biopsies with renal allograft pyelonephritis and allograft rejection. Clin. Transplant..

[20] Geiss GK (2008). Direct multiplexed measurement of gene expression with color-coded probe pairs. Nat. Biotechnol..

[21] Malkov VA (2009). Multiplexed measurements of gene signatures in different analytes using the Nanostring nCounter assay system. BMC Res. Notes.

[22] Hyeon J (2017). NanoString nCounter® approach in breast cancer: a comparative analysis with quantitative real-time polymerase chain reaction. J. Breast Cancer.

[23] Sigdel T (2019). Assessment of 19 genes and validation of CRM gene panel for quantitative transcriptional analysis of molecular rejection and inflammation in archival kidney transplant biopsies. Front Med (Lausanne).

[24] Venner JM, Hidalgo LG, Famulski KS, Chang J, Halloran PF (2015). The molecular landscape of antibody-mediated kidney transplant rejection: evidence for NK involvement through CD16a Fc receptors. Am. J. Transplant..

[25] Madill-Thomsen KS, Wiggins RC, Eskandary F, Böhmig GA, Halloran PF (2017). The effect of cortex/medulla proportions on molecular diagnoses in kidney transplant biopsies: rejection and injury can be assessed in medulla. Am. J. Transplant..

[26] Wang Z, Lyu Z, Pan L, Zeng G, Randhawa P (2019). Defining housekeeping genes suitable for RNA-seq analysis of the human allograft kidney biopsy tissue. BMC Med. Genom..

[27] Aggerholm-Pedersen N (2014). The importance of reference gene analysis of formalin-fixed, paraffin-embedded samples from sarcoma patients—an often underestimated problem. Transl. Oncol..

[28] Loupy A (2017). The Banff 2015 kidney meeting report: current challenges in rejection classification and prospects for adopting molecular pathology. Am. J. Transplant..

[29] Haas M (2018). The Banff 2017 kidney meeting report: revised diagnostic criteria for chronic active T cell-mediated rejection, antibody-mediated rejection, and prospects for integrative endpoints for next-generation clinical trials. Am. J. Transplant..

[30] Livak KJ, Schmittgen TD (2001). Analysis of relative gene expression data using real-time quantitative PCR and the 2(-delta delta C(T)) method. Methods.

