# Supplementary material for: Technical considerations when designing a gene expression panel for renal transplant diagnosis
Source: Sci Rep. 2020 Oct 21;10:17909. doi: 10.1038/s41598-020-74794-3 (PMC7578804; doi:10.1038/s41598-020-74794-3)
Supplement: Supplementary file 1 — Supplementary Figure. [file 41598_2020_74794_MOESM1_ESM.pdf]

TECHNICAL CONSIDERATIONS WHEN DESIGNING A GENE EXPRESSION PANEL FOR RENAL TRANSPLANT DIAGNOSIS

F Toulza, K Dominy, T Cook, J Galliford, J Beadle, A McLean , C Roufosse

Supplemental Figure 1

Overall correlation of gene expression between Nanostring and qRT-PCR

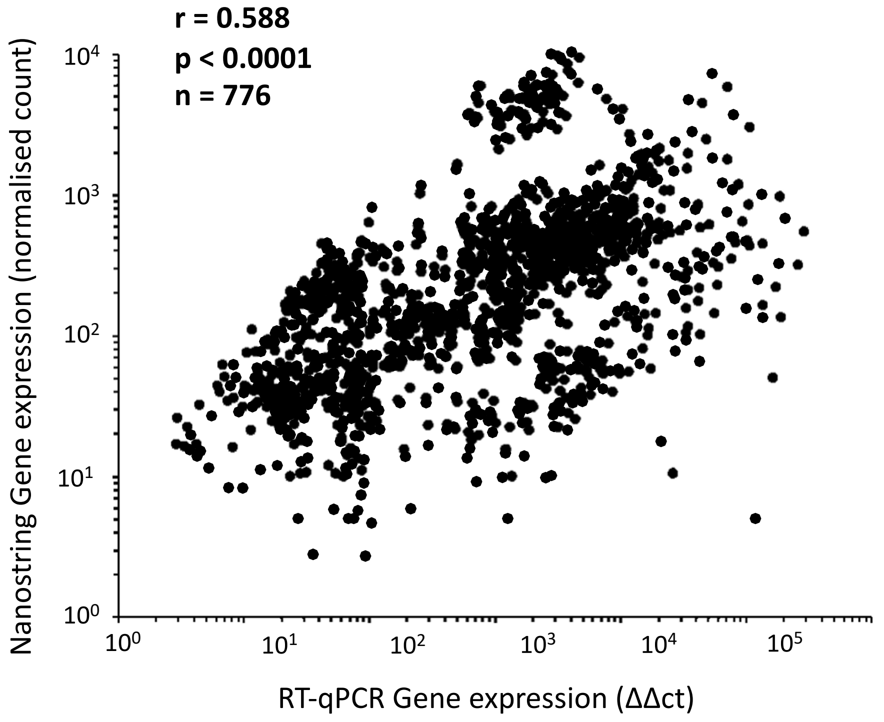

Individual gene expression correlation between Nanostring and qRT-PCR

	Spearman Test	
**Gene**	Correlation coef.	p value
CCL4	0.904	<0.001
CXCL10	0.845	<0.001
GNLY	0.775	<0.001
CXCL11	0.764	<0.001
VWF	0.709	<0.001
CD160	0.705	<0.001
SH2D1B	0.703	<0.001
DARC	0.695	<0.001
ROBO4	0.619	<0.001
CDH5	0.574	<0.001
MYBL1	0.504	<0.001
KLRF1	0.502	<0.001
FGFBP2	0.477	0.005
PLA1A	0.461	0.001
PECAM1	0.459	0.001
KLF4	0.337	0.016
SOX7	0.262	0.03
**CX3CR1**	0.26	0.229

NanoString vs. qRT-PCR gene expression quantification. The scatter plot shows expression results for up to 18 genes in 51 samples as quantified by qRT-PCR and NanoString on corresponding RNA later preserved biopsy. (only plot when both value in Nanostring and qRT-PCR have been measured)
