# Supplementary material for: Technical considerations when designing a gene expression panel for renal transplant diagnosis
Source: Sci Rep. 2020 Oct 21;10:17909. doi: 10.1038/s41598-020-74794-3 (PMC7578804; doi:10.1038/s41598-020-74794-3)
Supplement: Supplementary file 2 — Supplementary Table 1. [file 41598_2020_74794_MOESM2_ESM.pdf]

TECHNICAL CONSIDERATIONS WHEN DESIGNING A GENE EXPRESSION PANEL FOR RENAL TRANSPLANT DIAGNOSIS

F Toulza, K Dominy, T Cook, J Galliford, J Beadle, A McLean , C Roufosse

Supplemental Table S1: Spearman Rank correlation values for 74 AMR-related genes compared in 51 biopsies between FFPE and RNAlater samples using the NanoString platform

(A) Genes with significant correlation

Gene	Spearman correlation	P value
FCGR3A	.879**	0.000
GZMB	.859**	0.000
CCL4	.848**	0.000
CXCL11	.837**	0.000
TRD	.833**	0.000
CXCL10	.831**	0.000
PSMB10	.788**	0.000
CXCL9	.785**	0.000
IFI27	.778**	0.000
TBX21	.777**	0.000
CYP4F11	.768**	0.000
ICAM1	.752**	0.000
CX3CR1	.749**	0.000
KLRF1	.737**	0.000
TNF	.715**	0.000
DARC	.701**	0.000
CCL3	.686**	0.000
CD74	.685**	0.000
CETP	.683**	0.000
SELE	.681**	0.000
CD160	.657**	0.000
IFNG	.639**	0.000
APOL1	.627**	0.000
GNLY	.616**	0.000

Gene	Spearman correlation	P value
PLA1A	.551**	0.000
FGFBP2	.540**	0.000
ICAM2	.507**	0.000
MYBL1	.493**	0.000
VWF	.472**	0.000
EVA1C	.471**	0.000
S1PR5	.464**	0.000
SH2D1B	.451**	0.000
THBD	.435**	0.001
RPS6	.435**	0.001
ECSCR	.430**	0.001
TEK	.412**	0.002
CD55	.389**	0.004
CD59	.387**	0.005
KLF4	.381**	0.006
MMRN1	.367**	0.008
PECAM1	.347*	0.012
RAMP3	.347*	0.013
SOST	.334*	0.016
CDH13	.327*	0.019
NPHS1	.299*	0.033
NPHS2	.290*	0.038
CX3CL1	.291*	0.039
TRDV3	.288*	0.040

TRIB1	.606**	0.000	*CD34*	.279*	0.041

(B) Genes without significant correlation

Gene	Spearman correlation	P value
EHD3	0.246	0.081
MEOX1	0.233	0.099
APOBEC3A	0.231	0.102
TM4SF18	0.219	0.122
RAPGEF5	0.205	0.149
TM4SF1	0.196	0.166
MALL	0.188	0.185
COL4A3	0.188	0.186
CAV1	0.178	0.209
COL13A1	0.160	0.256
RASIP1	0.129	0.364
ROBO4	0.129	0.363
CDH5	0.127	0.374
GATA3	0.093	0.512
GNG11	0.092	0.519
PLAT	0.087	0.542
PGM5	0.082	0.567
COL4A5	0.046	0.743
COL4A5	0.016	0.910
PALMD	0.003	0.980
SOX7	0.002	0.987
RHOJ	-0.006	0.968
S1PR1	-0.005	0.680
RPS6KB1	-0.110	0.441
