# Supplementary material for: Technical considerations when designing a gene expression panel for renal transplant diagnosis
Source: Sci Rep. 2020 Oct 21;10:17909. doi: 10.1038/s41598-020-74794-3 (PMC7578804; doi:10.1038/s41598-020-74794-3)
Supplement: Supplementary file 3 — Supplementary Table 2. [file 41598_2020_74794_MOESM3_ESM.pdf]

TECHNICAL CONSIDERATIONS WHEN DESIGNING A GENE EXPRESSION PANEL FOR RENAL TRANSPLANT DIAGNOSIS

F Toulza, K Dominy, T Cook, J Galliford, J Beadle, A McLean , C Roufosse

Supplemental Table S2: Effect of Gene Level of Expression on the Correlation Between FFPE and RNAlater (RL) Samples.

	Number of genes with significant correlation between FFPE and RL (percentage)	Number of genes with non-significant correlation between FFPE and RL (percentage)
Group 1: Low Expression (0-100)	43 (82.7%)	9 (17.9%)
Group 2 :Intermediate expression (100-1000)	82 (80%)	22 (20%)
Group 3 : High expression (>1000)	46 (72%)	16 (28%)
**ALL genes**	**173 (78.6%)**	**47 (21.4%)**

Effect of gene Level of expression on the correlation between FFPE and RL samples

The 219 genes cover a wide range of absolute count levels on NanoString, from 4.67 to 137,352.5 for FFPE, and from 5.73 to 140,512.2 for RNAlater. To test the hypothesis that poor correlation between FFPE and RNAlater samples may be related to the level of expression of the genes, we divided the 219 genes into 3 groups according to the geometric mean (Geomean) of the expression levels over the 51 samples in both FFPE and RL. The level of expression for each gene corresponds to the mean expression measured by NanoString after normalization. Group 1 ("low") comprised those with a Geomean <100, Group 2 ("intermediate") comprised Geomean values of 100-1000, and Group 3 ("high") comprised those with a Geomean > 1000. We then compared the proportion of non-significant genes in each group. Numbers of genes in each group are presented for significant and non-significant correlations, followed by percentage in brackets. Analysis using the Chi-square test showed no significant difference between the 3 groups.
