# Supplementary material for: Technical considerations when designing a gene expression panel for renal transplant diagnosis
Source: Sci Rep. 2020 Oct 21;10:17909. doi: 10.1038/s41598-020-74794-3 (PMC7578804; doi:10.1038/s41598-020-74794-3)
Supplement: Supplementary file 4 — Supplementary Table 3. [file 41598_2020_74794_MOESM4_ESM.pdf]

TECHNICAL CONSIDERATIONS WHEN DESIGNING A GENE EXPRESSION PANEL FOR RENAL TRANSPLANT DIAGNOSIS

F Toulza, K Dominy, T Cook, J Galliford, J Beadle, A McLean , C Roufosse

Supplemental Table S3: Spearman Rank correlation values for 11 House Keeping genes compared in 51 biopsies between FFPE and RNAlater samples using the NanoString platform

(A) House Keeping Genes with significant correlation

Gene	Spearman correlation	P value
SDHA	.690**	0.000
GUSB	.474**	0.000
ACTB	.419**	0.000
LDHA	.317**	0.023
DDX50	.283**	0.043
HPRT1	.275*	0.045

(B) House Keeping Genes without significant correlation

Gene	Spearman correlation	P value
HDAC3	0.237	0.093
UBB	0.137	0.335
GAPDH	0.115	0.419
POLR2A	0.083	0.558
OAZ1	-0.041	0.769
