# Supplementary material for: Technical considerations when designing a gene expression panel for renal transplant diagnosis
Source: Sci Rep. 2020 Oct 21;10:17909. doi: 10.1038/s41598-020-74794-3 (PMC7578804; doi:10.1038/s41598-020-74794-3)
Supplement: Supplementary file 5 — Supplementary Table 4. [file 41598_2020_74794_MOESM5_ESM.pdf]

TECHNICAL CONSIDERATIONS WHEN DESIGNING A GENE EXPRESSION PANEL FOR RENAL TRANSPLANT DIAGNOSIS

F Toulza, K Dominy, T Cook, J Galliford, J Beadle, A McLean , C Roufosse

Supplemental Table S4: Supplemental Table S4. Genes and primer sequences analysed in qRT-PCR experiments

Gene	Primers
HPRT1<br>*Hypoxanthine phosphoribosyltransferase 1*	5'-TGAGGATTTGGAAAGGGTGT<br>5'-AATCCAGCAGGTCAGCAAAG
CCL4<br>*Chemokine (C-C motif) ligand 4*	5'-GCTTCCTCGCAACTTTGTGG<br>5'-GGATTCACTGGGATCAGCACA
CD160<br>*CD160 antigen*	5'-TGCCAGAAGCCAGAAGTCAG<br>5'-AAGCCTGAACTGAGAGTGCC
CDH5<br>*Cadherin 5*	5'-ACCAGGACGCTTTCACCATT<br>5'-AGGGCTCATGTATCGGAGGT
CX3CR1<br>*chemokine (C-X3-C motif) receptor 1*	5'-AAGTCTGAGCAGGACAGGGT<br>5'-GATCCATGGTGAAGGCCTGG
CXCL10<br>*C-X-C motif chemokine ligand 10*	5'-AGCAGAGGAACCTCCAGTCT<br>5'-ATGCAGGTACAGCGTACAGT
CXCL11<br>*C-X-C motif chemokine ligand 11*	5'-ATAGGCCCTGGGGTAAAAGC<br>5'-CTTGCTTGCTTCGATTTGGGA
DARC<br>*Duffy Coat Antigen Receptor*	5'-TGACAGCCGTCCCAGCCCTT<br>5'-AGAGCTCCGCCCTGTGCAGA
FGFBP2<br>*Fibroblast Growth Factor binding Protein 2*	5'-AGAAGAAGGCCTGGGAACAT<br>5'-TTCCAGTGTGAGAACGTTGG
GNLY<br>*Granulysin*	5'-TGACCAAAACACAGGAGCTG<br>5'-GATCTGCTGGGCAGTTTCTC
KLF4<br>*Kruppel like factor 4*	5'-CACACTTGTGATTACGCGGG<br>5'-GCGAATTTCCATCCACAGCC
KLRF1<br>*Killer Cell Lectin-like receptor subfamily F, Member 1*	5'-GCCACTCAGTATGAGGACACT<br>5'-CCCTTGGTATTTGAGCCATTCTG
MLYB1<br>*v-myb myeloblastosis viral oncogene homolog (avian) like 1*	5'-TGAGGATGAGGATGATGACC<br>5'-CCAATCATCAGTTCCATGTTG
PECAM1<br>*Platelet / Endothelial cell Adhesion Molecule 1*	5'-CCACTGCAGAGTACCAGGTG<br>5'-CCACCTTGGATGGCCTCTTT
PLA1a<br>*phospholipase A1 member A*	5'-CAGCCTCGTGGAGTTTCACT<br>5'-GGGTGGCATGGGCTATGATT
ROBO4<br>*Roundabout guidance receptor 4*	5'-GAAGTCAGCTCCACTGTCGT<br>5'-AGGGAGAACTCTCTGGAGGC
SH2D1b (EAT2)<br>*SH2 domain containing 1B*	5'-CGAATCTTCAGAGAGAAACACG<br>5'-GGGCTGGTTCTCTTTATTGG
SOX7<br>*Sex determining region Y box 7*	5'-GACGAGAGGAAACGGCTGG<br>5'-TACGGCCTCTTCTGGGACAG
vWF<br>*von Willebrand Factor*	5'-AGATGTTTGCCTACGGCTTG<br>5'-CAGCCTGTGACCCTCTTCTC
