# Supplementary material for: Technical considerations when designing a gene expression panel for renal transplant diagnosis
Source: Sci Rep. 2020 Oct 21;10:17909. doi: 10.1038/s41598-020-74794-3 (PMC7578804; doi:10.1038/s41598-020-74794-3)
Supplement: Supplementary file 6 — Supplementary Table 5. [file 41598_2020_74794_MOESM6_ESM.pdf]

TECHNICAL CONSIDERATIONS WHEN DESIGNING A GENE EXPRESSION PANEL FOR RENAL TRANSPLANT DIAGNOSIS

F Toulza, K Dominy, T Cook, J Galliford, J Beadle, A McLean , C Roufosse

Supplemental Table S5: **Nanostring Custom Codeset of 219 selected genes**

Gene Identifier	Accession	Position	Target Sequence
ACTB	NM_001101.2	1011-1110	TGCAGAAGGAGATCACTGCCCTGGCACCCAGCACAATGAAGATCAAGATCATTGCTCCT CCTGAGCGCAAGTACTCCGTGTGGATCGGCGGCTCCATCCT
ADAMDEC1	NM_014479.2	907-1006	TTGACAACTTCCTGAGATGGCACAGTTCTAACCTGGGGAAAAAGATCCACGACCATGCT CAGCTTCTCAGCGGGATTAGCTTCAACAATCGACGTGTGGG
ADB15	NM_080928.3	1029-1128	AAATGGTTTTGATGTCAACACTCTACTTGCTGACCACATTTCCCAGAGCTATGACGATGA GAGGAAGACTGCGCTGTATTTTGGCGTTTCTAATAATGAC
AIM2	NM_004833.1	608-707	ACGTGCTGCACCAAAAGTCTCTCCTCATGTTAAGCCTGAACAGAAACAGATGGTGGCCC AGCAGGAATCTATCAGAGAAGGGTTTCAGAAGCGCTGTTTG
AKT1 (PKB-alpha)	NM_001014432.1	1276-1375	GGACGGGCACATTAAGATCACAGACTTCGGGCTGTGCAAGGAGGGGATCAAGGACGGT GCCACCATGAAGACCTTTTGCGGCACACCTGAGTACCTGGCC
AKT2 (PKB-beta)	NM_001626.4	700-799	AGGAGATGGAAGTGGCGGTCAGCAAGGCACGGGCTAAAGTGACCATGAATGACTTCGA CTATCTCAAACTCCTTGGCAAGGGAACCTTTGGCAAAGTCAT
AKT3 (PKB-gamma)	NM_005465.4	288-387	GCCAGTTAATGAAAACAGAACGACCAAAGCCAAACACATTTATAATCAGATGTCTCCAGT GGACTACTGTTATAGAGAGAACATTTCATGTAGATACTCC
ANKRD22	NM_144590.2	58-157	ACTGGCAGAGCAAATATGACTCAGAAACCGGCTCCTCAGGGTTGTAACATTAGATGATA CAGGCTTGGGTCGTTACACATGACACCAGTGCCTTTGTTTC
AOAH	NM_001637.3	1304-1403	CTCTGGTGCTACAGGATTTCTGGACTCCACTGTTGGAATTAAAGAAAAATCTATTTACCTT CGCTTATGGAAAAGAAACCACTGTAATCACAGGGACTAC
AP-1/JUN	NM_002228.3	141-240	ACACAGCCAGCCAGCCAGGTCGGCAGTATAGTCCGAACTGCAAATCTTATTTTCTTTTCA CCTTCTCTCTAACTGCCCAGAGCTAGCGCCTGTGGCTCCC
APOBEC3A	NM_145699.3	32-131	CAGCAGCTTCCAGGTTGCTCTGATGATATATTAAGGCTCCTGAATCCTAAGAGAATGTTG GTGAAGATCTTAACACCACGCCTTGAGCAAGTCGCAAGAG
APOL1	NM_003661.3	821-920	CCGCTTTGACCGGGATTACCAGCAGTACCATGGACTACGGAAAGAAGTGGTGGACACAA GCCCAAGCCCACGACCTGGTCATCAAAAGCCTTGACAAATT
APOL2	NM_030882.2	881-980	GCAACTTGGACCAAAGCGGCACCAATGTAGCAAAGGTGATGAAGGAGTTTGTGGGTGG GAACACACCCAATGTTCTTACCTTAGTTGACAATTGGTACCA

BASP1	NM_006317.3	1161-1260	GATCCGCGTCTGAAAGTGCAGTACATCGTTTGTACCTGAAACTGCCGCCACATGCACTCCTCCACCGCTGAGAGTTGAATAGCTTTTCTTCTGCAATGGG
BCL2	NM_000657.2	6-105	GTGAAGCAGAAGTCTGGGAATCGATCTGGAAATCCTCCTAATTTTTACTCCCTCTCCCCGCGACTCCTGATTCATTGGGAAGTTTCAAATCAGCTATAAC
BLNK	NM_013314.2	931-1030	ACACCACTGAAGACAACTCCAGTTGCCTCTCAACAGAATGCTTCAAGTGTTTGTGAAGAAAAACCTATACCTGCTGAACGCCACCGAGGGTCAAGTCACA
BTK	NM_000061.1	571-670	TGATCTGGTTCAGAAATATCACCCTTGCTTCTGGATCGATGGGCAGTATCTCTGCTGCTCTCAGACAGCCAAAAATGCTATGGGCTGCCAAATTTTGGAG
BTLA	NM_181780.2	306-405	ACACTCCATCTTAGCAGGAGATCCCTTTGAACTAGAATGCCCTGTGAAATACTGTGCTAACAGGCCTCATGTGACTTGGTGCAAGCTCAATGGAACAACA
CAV1	NM_001753.3	435-534	AACCGCGACCCTAAACACCTCAACGATGACGTGGTCAAGATTGACTTTGAAGATGTGATTGCAGAACCAGAAGGGACACACAGTTTTGACGGCATTTGGA
CCL2	NM_002982.3	1-100	GAGGAACCGAGAGGCTGAGACTAACCCAGAAACATCCAATTCTCAAACTGAAGCTCGCACTCTCGCCTCCAGCATGAAAGTCTCTGCCGCCCTTCTGTGC
CCL3	NM_002983.2	682-781	CTGTGTAGGCAGTCATGGCACCAAAGCCACCAGACTGACAAATGTGTATCGGATGCTTTTGTTCAGGGCTGTGATCGGCCTGGGGAAATAATAAAGATGC
CCL4	NM_002984.2	202-301	GAAGCTTCCTCGCAACTTTGTGGTAGATTACTATGAGACCAGCAGCCTCTGCTCCCAGCCAGCTGTGGTATTCCAAACCAAAAGAAGCAAGCAAGTCTGT
CCL5	NM_002985.2	281-380	AGTGTGTGCCAACCCAGAGAAGAAATGGGTTCGGGAGTACATCAACTCTTTGGAGATGAGCTAGGATGGAGAGTCCTTGAACCTGAACTTACACAAATTT
CCR5	NM_000579.1	2731-2830	TAGGAACATACTTCAGCTCACACATGAGATCTAGGTGAGGATTGATTACCTAGTAGTCATTTCATGGGTTGTTGGGAGGATTCTATGAGGCAACCACAGG
CD160	NM_007053.3	723-822	AACAAAGACAACACCTTGAGTTCAGCCATAATGAAGGCACTCTCAGTTCAGGCTTCCTACAAGAAAAGGTCTGGGTAATGCTGGTCACCAGCCTTGTGGC
CD25	NM_000417.1	1001-1100	CTTGGTAAGAAGCCGGGAACAGACAACAGAAGTCATGAAGCCCAAGTGAAATCAAAGGTGCTAAATGGTCGCCCAGGAGACATCCGTTGTGCTTGCCTGC
CD274	NM_014143.3	50-149	AGCTTCCCGAGGCTCCGCACCAGCCGCGCTTCTGTCCGCCTGCAGGGCATTCCAGAAAGATGAGGATATTTGCTGTCTTTATATTCATGACCTACTGGCA
CD28	NM_001243078.1	2066-2165	GCTGCTCCTGTACCTTGGAGGTCCATTCACATGGGAAAGTATTTTGGAATGTGTCTTTTGAAGAGAGCATCAGAGTTCTTAAGGGACTGGGTAAGGCCTG
CD34	NM_001025109.1	1581-1680	AGGTAAACTCCTGTCCTTTACACATTCGGCTCCCTGGAGCCAGACTCTGGTCTTCTTTGGGTAAACGTGTGACGGGGGAAAGCCAAGGTCTGGAGAAGCT
CD3D	NM_000732.4	111-210	TATCTACTGGATGAGTTCCGCTGGGAGATGGAACATAGCACGTTTCTCTCTGGCCTGGTACTGGCTACCCTTCTCTCGCAAGTGAGCCCCTTCAAGATAC
CD4	NM_000616.4	976-1075	TGGCAGGCGGAGAGGGCTTCCTCCTCCAAGTCTTGGATCACCTTTGACCTGAAGAACAAGGAAGTGTCTGTAAAACGGGTTACCCAGGACCCTAAGCTCC
CD55	NM_000574.3	1130-1229	CACCACCTGAATGCAGAGGAAAATCTCTAACTTCCAAGGTCCCACCAACAGTTCAGAAACCTACCACAGTAAATGTTCCAACTACAGAAGTCTCACCAAC

CD59	NM_000611.4	731-830	GACTTGAACTAGATTGCATGCTTCCTCCTTTGCTCTTGGGAAGACCAGCTTTGCAGTGAC AGCTTGAGTGGGTTCTCTGCAGCCCTCAGATTATTTTTCC
CD6	NM_001254751.1	1723-1822	AACCCTGGACACTGCATTACAGACCCGCCATCCCTGGGCCCTCAGTATCACCCGAGGA GCAACAGTGAGTCGAGCACCTCTTCAGGGGAGGATTACTGCA
CD7	NM_006137.6	441-540	CCTACACCTGCCAGGCCATCACGGAGGTCAATGTCTACGGCTCCGGCACCCTGGTCCT GGTGACAGAGGAACAGTCCCAAGGATGGCACAGATGCTCGGA
CD72	NM_001782.2	453-552	CCGCCATCTGCCTGGGAGTGCGCTATCTGCAGGTGTCTCAGCAGCTCCAGCAGACGAA CAGGGTTCTGGAAGTCACTAACAGCAGCCTGAGGCAGCAGCT
CD74	NM_001025159.1	965-1064	TTCAGCCCCCAGCCCCTCCCCCATCTCCCACCCTGTACCTCATCCCATGAGACCCTGGT GCCTGGCTCTTTCGTCACCCTTGGACAAGACAAACCAAGTC
CD84	NM_001184879.1	29-128	TCTGCTAGAACAGTGCCGTGCTTTTCCACAGAAGGTTAGACCCTGAAAGAGATGGCTCA GCACCACCTATGGATCTTGCTCCTTTGCCTGCAAACCTGGC
CD86	NM_175862.3	1266-1365	CCAGCTCTGCTCCGTATGCCAAGAGGAGACTTTAATTCTCTTACTGCTTCTTTTCACTTCA GAGCACACTTATGGGCCAAGCCCAGCTTAATGGCTCATG
CD8A	NM_001768.5	1321-1420	GCTCAGGGCTCTTTCCTCCACACCATTCAGGTCTTTCTTTCCGAGGCCCCTGTCTCAGG GTGAGGTGCTTGAGTCTCCAACGGCAAGGGAACAAGTACTT
CD8B	NM_172099.2	440-539	TCAGCTGAGTGTGGTTGATTTCCTTCCCACCACTGCCCAGCCCACCAAGAAGTCCACCC TCAAGAAGAGAGTGTGCCGGTTACCCAGGCCAGAGACCCAG
CD96	NM_005816.4	429-528	TCACTTGTGACTTTCACAGAAACTCCTGAGAATGGGTCAAAATGGACTCTGCACTTAAGG AATATGTCTTGTTCAGTCAGTGGAAGGTACGAGTGTATGC
CDH13	NM_001220488.1	211-310	CTCCTCAAAGCCTGGCTCCCACGGAAAATATGCTCAGTGCAGCCGCGTGCATGAATGAA AACGCCGCCGGGCGCTTCTAGTCGGACAAAATGCAGCCGAG
CDH5	NM_001795.3	3406-3505	TCTCCCCTTCTCTGCCTCACCTGGTCGCCAATCCATGCTCTCTTTCTTTTCTCTGTCTACT CCTTATCCCTTGGTTTAGAGGAACCCAAGATGTGGCCTT
CETP	NM_000078.2	663-762	AGAGATCAACGTCATCTCTAACATCATGGCCGATTTTGTCCAGACAAGGGCTGCCAGCA TCCTTTCAGATGGAGACATTGGGGTGGACATTTCCCTGACA
CHCHD10	NM_213720.1	369-468	CTGCAGATGGGGCCCTGCGCCTACGAGATCAGGCAGTTCCTGGACTGTTCCACCACTC AGAGTGACCTGTCCCTGTGTGAGGGCTTCAGCGAGGCCCTGA
COL13A1	NM_080798.3	1658-1757	CAAGGGCTCCAAGGGAGAACCAGGGAAAGGAGAGATGGTGGATTACAATGGAAACATC AATGAGGCTCTCCAGGAGATCCGGACGCTGGCCTTGATGGGG
COL1A1	NM_000088.3	5211-5310	CAGAAACATCGGATTTGGGGAACGCGTGTCAATCCCTTGTGCCGCAGGGCTGGGCGGG AGAGACTGTTCTGTTCCTTGTGTAACTGTGTTGCTGAAAGAC
COL4A3	NM_000091.4	1085-1184	CTGGAAGTGAGGGAGTCAAGGGCAACAGGGGTTTCCCTGGGTTAATGGGTGAAGATGG CATTAAGGGACAGAAAGGGGACATTGGCCCTCCAGGATTTCG
COL4A4	NM_000092.4	761-860	TATATGGGAGTGGAAAGAAATACATTGGTCCTTGTGGAGGAAGAGATTGCTCTGTTTGC CACTGTGTTCCTGAAAAGGGGTCTCGGGGTCCACCAGGACC
COL4A5	NM_000495.4	1715-1814	GTGACACTTGCTTCAACTGCATTGGAACTGGTATTTCAGGGCCTCCAGGTCAACCTGGT TTGCCAGGTCTCCCAGGTCCTCCAGGATCTCTTGGTTTCCC

CPA3	NM_001870.2	221-320	ACCCACCACGTAGCTGCTAATATGATGGTGGATTTCCGAGTTAGTGAGAAGGAATCCCA AGCCATCCAGTCTGCCTTGGATCAAAATAAAATGCACTATG
CTLA-4	NM_005214.3	406-505	AGTCTGTGCGGCAACCTACATGATGGGGAATGAGTTGACCTTCCTAGATGATTCCATCT GCACGGGCACCTCCAGTGGAAATCAAGTGAACCTCACTATC
CTSS	NM_004079.3	686-785	ATGACAACGGCTTTCCAGTACATCATTGATAACAAGGGCATCGACTCAGACGCTTCCTAT CCCTACAAAGCCATGGATCAGAAATGTCAATATGACTCAA
CX3CL1	NM_002996.3	1217-1316	CCCCGGAGCTGTGGTAGTAATTCATATGTCCTGGTGCCCGTGTGAACTCCTCTGGCCTG TGTCTAGTTGTTTGATTCAGACAGCTGCCTGGGATCCCTCA
CX3CR1	NM_001337.3	1041-1140	GGGCGCTCAGTCCACGTTGATTTCTCCTCATCTGAATCACAAAGGAGCAGGCATGGAAG TGTTCTGAGCAGCAATTTTACTTACCACACGAGTGATGGAG
CXCL1	NM_001511.1	743-842	TATGTTAATATTTCTGAGGAGCCTGCAACATGCCAGCCACTGTGATAGAGGCTGGCGGA TCCAAGCAAATGGCCAATGAGATCATTGTGAAGGCAGGGGA
CXCL10	NM_001565.2	462-561	GCCATAATTGTTCTTAGTTTGCAGTTACACTAAAAGGTGACCAATGATGGTCACCAAATC AGCTGCTACTACTCCTGTAGGAAGGTTAATGTTCATCATC
CXCL11	NM_005409.4	283-382	TTCAAAAGAGGACGCTGTCTTTGCATAGGCCCTGGGGTAAAAGCAGTGAAAGTGGCAGA TATTGAGAAAGCCTCCATAATGTACCCAAGTAACAACTGTG
CXCL13	NM_006419.2	211-310	AGACGCTTCATTGATCGAATTCAAATCTTGCCCCGTGGGAATGGTTGTCCAAGAAAAGAA ATCATAGTCTGGAAGAAGAACAAGTCAATTGTGTGTGTGG
CXCL9	NM_002416.1	1976-2075	CACCATCTCCCATGAAGAAAGGGAACGGTGAAGTACTAAGCGCTAGAGGAAGCAGCCA AGTCGGTTAGTGGAAGCATGATTGGTGCCCAGTTAGCCTCTG
CXCR1	NM_000634.2	1951-2050	GCAGCCACCAGTCCATTGGGCAGGCAGATGTTCCTAATAAAGCTTCTGTTCCGTGCTTG TCCCTGTGGAAGTATCTTGGTTGTGACAGAGTCAAGGGTGT
CXCR6	NM_006564.1	96-195	TTACCATGAAGACTATGGGTTCAGCAGTTTCAATGACAGCAGCCAGGAGGAGCATCAAG ACTTCCTGCAGTTCAGCAAGGTCTTTCTGCCCTGCATGTAC
CYP4F11	NM_021187.3	3201-3300	TCATAGCCTAAGGGATGTTGCATCGCTAAACCCTGTTGACTTCTCTAATCTCTTCTGTAG CTGATCTCCTTCCCTTGCTACACTCGGTTACTCTGGCCTT
DARC	NM_002036.2	961-1060	CCCTGGATGAATATCCTGTGGGCCTGGTTTATTTTCTGGTGGCCTCATGGGGTGGTTCT AGGACTGGATTTCCTGGTGAGGTCCAAGCTGTTGCTGTTGT
DDX50	NM_024045.1	1186-1285	CTACTGTGGAACATTTGGCCATCCAGTGTCATTGGTCTCAGAGGCCAGCAGTTATTGGA GATGTCCTTCAAGTCTACAGTGGGTCTGAAGGGAGGGCTAT
DUSP2	NM_004418.3	1236-1335	CTGGCCCTCATTCGGGGTCGGGAACCAAGGGTGTGTCTGCTCTTTCCCTCCCCATCCTC TGGCAGAAATCAGCTAGACGCTATACCGTGGACTCTCCCTG
ECSCR	NM_001077693.3	169-268	TAGCTCTCAGGGAGGCCTTGGCGGTCTAAGTCTGACCACAGAGCCAGTTTCTTCCAACC CAGGATACATCCCTTCCTCAGAGGCTAACAGGCCAAGCCAT
EHD3	NM_014600.2	2327-2426	CATATCTTGACATTGCTCTGTAGGTGAGAGAGGACCATGACGCCCATGTTTGCAGCTGA TACTTGTTTGGGCACACCTCCAAGTTCTCGGGATTAGAAGG
EVA1C	NM_058187.3	1155-1254	GTGAAAAAATACCTCACTGTGACCTACGCATGTGTTCCCAAGAACATACTCACAGCGATT GATCCAGCCATTGCTAATCTAAAACCTTCTTTGAAGCAGA

EZH2	NM_001203247.1	1122-1221	ACACAGAAACAGCTCTAGACAACAAACCTTGTGGACCACAGTGTTACCAGCATTTGGAG GGAGCAAAGGAGTTTGCTGCTGCTCTCACCGCTGAGCGGAT
FAM26F	NM_001010919.1	901-1000	AGGGCCAGTACTACAGCATGTTGCACAAATATGTCAACAGAAAAGAGAAGACTCACAGT ATCAGGTCTACTGAAGGAGATACGGTGATTCCTGTTCTTGG
FCGR1B	NM_001017986.3	499-598	ATTCTAACCTCACCATTCTGAAAACCAACATAAGTCACAATGGCACCTACCATTGCTCAG GCATGGGAAAGCATCGCTACACATCAGCAGGAATATCACA
FCGR3A	NM_000569.6	1645-1744	AAATCATGAGGGTGACGTAGAATTGAGTCTTCCAGGGGACTCTATCAGAACTGGACCAT CTCCAAGTATATAACGATGAGTCCTCTTAATGCTAGGAGTA
FGD2	NM_173558.3	1120-1219	TCTAACACCCTGCTCCGTGAGGGCCCGGTCCTCAAGATCTCCTTCCGCCGCAACGACC CCATGGAGCGCTACCTTTTCTTGTTCAACAACATGCTGCTCT
FGFBP2	NM_031950.3	952-1051	CTTTCTGGAGTTTGCAGAGTTCAGCAATATGATAGGGAACAGGTGCTGATGGGCCCAAG AGTGACAAGCATACACAACTACTTATTATCTGTAGAAGTTT
FJX1	NM_014344.3	2377-2476	TCATTCCTGACCTCTTGTCATTTTGGCCTGAAGGCTACAAATTCAGGGTCAGCTGTATGC ACTAAGTCAAATAATGAATTTCTTCCTCCCTCTCGCAACC
FoxP3	NM_014009.3	1231-1330	GGGCCATCCTGGAGGCTCCAGAGAAGCAGCGGACACTCAATGAGATCTACCACTGGTT CACACGCATGTTTGCCTTCTTCAGAAACCATCCTGCCACCTG
GAPDH	NM_001256799.1	387-486	GAACGGGAAGCTTGTCATCAATGGAAATCCCATCACCATCTTCCAGGAGCGAGATCCCT CCAAAATCAAGTGGGGCGATGCTGGCGCTGAGTACGTCGTG
GATA3	NM_001002295.1	1692-1791	GTGCATGACTCACTGGAGGACTTCCCCAAGAACAGCTCGTTTAACCCGGCCGCCCTCTC CAGACACATGTCCTCCCTGAGCCACATCTCGCCCTTCAGCC
GBP5	NM_052942.3	1956-2055	ATTACAGACTGACCAGGCTCTCACAGAGACGGAAAAAAAGAAGAAAGAGGCACAAGTGA AAGCAGAAGCTGAAAAGGCTGAAGCGCAAAGGTTGGCGGCG
GIMAP5	NM_018384.3	1277-1376	ATCTCTGGACCCTGGAGCACTTCTAATGTATCACCCCATGGAGTCATTGTTCTAATAATC ACCAATTCAGACTCAGATCCTCGTGGTCTATGGAGCATGC
GNG11	NM_004126.3	431-530	GAAAATGCCTGCCCTTCACATCGAAGATTTGCCAGAGAAGGAAAAACTGAAAATGGAAG TTGAGCAGCTTCGCAAAGAAGTGAAGTTGCAGAGACAACAA
GNLY	NM_012483.2	881-980	TGCCGGCTCCTCGCTTCCTCGATCCAGAATCCACTCTCCAGTCTCCCTCCCCTGACTCC CTCTGCTGTCCTCCCCTCTCACGAGAATAAAGTGTCAAGCA
GUSB	NM_000181.3	1900-1999	CCGATTTCATGACTGAACAGTCACCGACGAGAGTGCTGGGGAATAAAAAGGGGATCTTC ACTCGGCAGAGACAACCAAAAAGTGCAGCGTTCCTTTTGCG
GZMB	NM_004131.3	541-640	ACACTACAAGAGGTGAAGATGACAGTGCAGGAAGATCGAAAGTGCGAATCTGACTTACG CCATTATTACGACAGTACCATTGAGTTGTGCGTGGGGGACC
HAVCR1	NM_001099414.1	969-1068	GGGAATGACACCGTGACAGAGTCTTCAGATGGCCTTTGGAATAACAATCAAACTCAACT GTTCCTAGAACATAGTCTACTGACGGCCAATACCACTAAAG
HDAC3	NM_003883.3	353-452	TGTTTCCCGGGCTCTTTGAGTTCTGCTCGCGTTACACAGGCGCATCTCTGCAAGGAGCA ACCCAGCTGAACAACAAGATCTGTGATATTGCCATTAACTG
HPRT1	NM_000194.1	241-340	TGTGATGAAGGAGATGGGAGGCCATCACATTGTAGCCCTCTGTGTGCTCAAGGGGGGC TATAAATTCTTTGCTGACCTGCTGGATTACATCAAAGCACTG

ICAM1	NM_000201.2	2254-2353	AAATACTGAAACTTGCTGCCTATTGGGTATGCTGAGGCCCCACAGACTTACAGAAGAAG TGGCCCTCCATAGACATGTGTAGCATCAAAACACAAAGGCC
ICAM2	NM_000873.3	416-515	ACCTCTCTAGATAAGATTCTGCTGGACGAACAGGCTCAGTGGAAACATTACTTGGTCTCA AACATCTCCCATGACACGGTCCTCCAATGCCACTTCACCT
ICOS	NM_012092.2	641-740	AACTCTGGCACCCAGGCATGAAGCACGTTGGCCAGTTTTCCTCAACTTGAAGTGCAAGA TTCTCTTATTTCCGGGACCACGGAGAGTCTGACTTAACTAC
IDO1	NM_002164.3	51-150	CTATTATAAGATGCTCTGAAAACTCTTCAGACACTGAGGGGCACCAGAGGAGCAGACTA CAAGAATGGCACACGCTATGGAAAACTCCTGGACAATCAGT
IFI27	NM_005532.3	391-490	TCACTGGGAGCAACTGGACTCTCCGGATTGACCAAGTTCATCCTGGGCTCCATTGGGTC TGCCATTGCGGCTGTCATTGCGAGGTTCTACTAGCTCCCTG
IFI30	NM_006332.3	386-485	CAGGAACAAAATGTCAGTGGCAGGTGGGAGTTCAAGTGCCAGCATGGAGAAGAGGAGT GCAAATTCAACAAGGTGGAGGCCTGCGTGTTGGATGAACTTG
IFNG	NM_000619.2	971-1070	ATACTATCCAGTTACTGCCGGTTTGAAAATATGCCTGCAATCTGAGCCAGTGCTTTAATG GCATGTCAGACAGAACTTGAATGTGTCAGGTGACCCTGAT
IKZF3	NM_183232.2	1177-1276	ATGTGTATCGGTGTGACCACTGCCGCGTCCTCTTCCTGGACTATGTGATGTTCACGATTC ACATGGGCTGCCACGGCTTCCGTGACCCTTTCGAGTGTAA
IL-1b	NM_000576.2	841-940	GGGACCAAAGGCGGCCAGGATATAACTGACTTCACCATGCAATTTGTGTCTTCCTAAAG AGAGCTGTACCCAGAGAGTCCTGTGCTGAATGTGGACTCAA
IL-6	NM_000600.3	365-464	GGCACTGGCAGAAAACAACCTGAACCTTCCAAAGATGGCTGAAAAAGATGGATGCTTCC AATCTGGATTCAATGAGGAGACTTGCCTGGTGAAAATCATC
IL12RB1	NM_005535.1	977-1076	AAGATGCCCTATCTCTCGGGTGCTGCCTACAACGTGGCTGTCATCTCCTCGAACCAATTT GGTCCTGGCCTGAACCAGACGTGGCACATTCCTGCCGACA
IL18BP	NM_001039659.1	1046-1145	CACAGCCTCCTTATAATGCCTCCTCCTCCTGCCATTCTCTCTCCACCTATCCATTAGCCT TCCTAACGTCCTACTCCTCACACTGCTCTACTGCTCAGAA
IL1RL1	NM_016232.4	701-800	CCTCTTGAGTGGTTTAAGAATTGTCAGGCTCTTCAAGGATCAAGGTACAGGGCGCACAA GTCATTTTTGGTCATTGATAATGTGATGACTGAGGACGCAG
IL21R	NM_181078.1	2730-2829	GGGCCACTCGGGGGGGTTTCCAGGCTTAAAATCAGTCCGTTTCGTCTCTTGGAAACAGC TCCCCACCAACCAAGATTTCTTTTTCTAACTTCTGCTACTA
IL23A	NM_016584.2	412-511	CAGGGACAACAGTCAGTTCTGCTTGCAAAGGATCCACCAGGGTCTGATTTTTTATGAGAA GCTGCTAGGATCGGATATTTTCACAGGGGAGCCTTCTCTG
IL8	NM_000584.2	26-125	ACAGCAGAGCACACAAGCTTCTAGGACAAGAGCCAGGAAGAAACCACCGGAAGGAACC ATCTCACTGTGTGTAAACATGACTTCCAAGCTGGCCGTGGCT
IMD52 (LAT)	NM_001014987.1	1291-1390	TGTGTAATAGAATAAAGGCCTGCGTGTGTCTGTGTTGAGCGTGCGTCTGTGTGTGCCTG TGTGCGAGTCTGAGTCAGAGATTTGGAGATGTCTCTGTGTG
INPP5D	NM_005541.3	4076-4175	ATAATGGCCACATGGATCGAACACTCATGATGTGCCAAGTGCTGTGCTAAGTGCTTTAC GAACATTCGTCATATCAGGATGACCTCGAGAGCTGAGGCTC
ITGB6	NM_000888.3	1091-1190	AACATTCTCCAGCTGATCATCTCAGCTTATGAAGAACTGCGGTCTGAGGTGGAACTGGA AGTATTAGGAGACACTGAAGGACTCAACTTGTCATTTACAG

KAAG1	NM_181337.3	19-118	AACATGCAGGGGGTGGGAGCAGGGTCACAAAAGTGAGTGTTGTCAATTCTACTTGGAATGAAAGGTTGAAATAATTTAAACAGTACGGGAAATGCAGAGC
KLF4	NM_004235.4	1981-2080	CGAGCATTTTCCAGGTCGGACCACCTCGCCTTACACATGAAGAGGCATTTTTAAATCCCAGACAGTGGATATGACCCACACTGCCAGAAGAGAATTCAGT
KLH13	NM_001168303.1	421-520	TCTCATCCCATTTGCAGTCTTCCAAGGCAGGACCTACACGCATCTTTACCAGCAATACCCACAGTTCTGTGGTGTTACAGGGCTTTGACCAGCTTCGACT
KLRF1	NM_016523.2	102-201	AGTCAAAGAAAAGGAGTTCTGCCCAAACATCTCAACTTACATTTAAAGATTATTCAGTGACGTTGCACTGGTATAAAATCTTACTGGGAATATCTGGAAC
LAG3	NM_002286.5	1736-1835	CTTTTGGTGACTGGAGCCTTTGGCTTTCACCTTTGGAGAAGACAGTGGCGACCAAGACGATTTTCTGCCTTAGAGCAAGGGATTCACCCTCCGCAGGCTC
LAIR1	NM_002287.3	1196-1295	GCACCTGAGGGTAGAAAGTCACTCTAGGAAAAGCCTGAAGCAGCCATTTGGAAGGCTTCCTGTTGGATTCCTCTTCATCTAGAAAGCCAGCCAGGCAGCT
LAP3	NM_015907.2	451-550	GTGCTAGTTGGCCTCGGCAAAAAGGCAGCTGGAATCGACGAACAGGAAAACTGGCATGAAGGCAAAGAAAACATCAGAGCTGCTGTTGCAGCGGGGTGCA
LCN2	NM_005564.3	326-425	AATGTCACCTCCGTCCTGTTTAGGAAAAAGAAGTGTGACTACTGGATCAGGACTTTTGTTCCAGGTTGCCAGCCCGGCGAGTTCACGCTGGGCAACATTA
LCP2	NM_005565.3	1828-1927	ATGAACCGTCCTCCTGCCTCTGTTGCCAACACGAGATCAATCAGCCTTGGTCAATGGACAAACACTTAGGACTGAACTGAACCCCTCCCCATGAACACAA
LDHA	NM_001165414.1	1691-1790	AACTTCCTGGCTCCTTCACTGAACATGCCTAGTCCAACATTTTTTCCCAGTGAGTCACATCCTGGGATCCAGTGTATAAATCCAATATCATGTCTTGTGC
LTF	NM_002343.2	591-690	TTCCCCAACCTGTGTCGCCTGTGTGCGGGGACAGGGGAAAACAAATGTGCCTTCTCCTCCCAGGAACCGTACTTCAGCTACTCTGGTGCCTTCAAGTGTC
MALL	NM_005434.3	606-705	ACAAGTACATGCCACGATTGTTTCTGAGAAACTGCTGGACCCAAGAATTTACTACATTAATTCGGCAGCCTCGTTCTTCGCCTTCATCGCCACGCTGCTC
MAPK1 (ERK)	NM_138957.2	431-530	ACTGCCAGAGAACCCTGAGGGAGATAAAAATCTTACTGCGCTTCAGACATGAGAACATCATTGGAATCAATGACATTATTCGAGCACCAACCATCGAGCA
MAPK11 (p38 b)	NM_002751.6	419-518	CACCCTGATGGGCGCCGACCTGAACAACATCGTCAAGTGCCAGGCGCTGAGCGACGAGCACGTTCAATTCCTGGTTTACCAGCTGCTGCGCGGGCTGAAG
MAPK12 / ERK6 (p38-g)	NM_002969.3	426-525	TATTCACTCCTGATGAGACCCTGGATGACTTCACGGACTTTTACCTGGTGATGCCGTTCATGGGCACCGACCTGGGCAAGCTCATGAAACATGAGAAGCT
MAPK13 /SAPK4 (p38-s)	NM_002754.3	1051-1150	CGGAGGCCCAGCAGCCGTTTGATGATTCCTTAGAACACGAGAAACTCACAGTGGATGAATGGAAGCAGCACATCTACAAGGAGATTGTGAACTTCAGCCC
MAPK14 (p38-a)	NM_001315.2	793-892	GGTGACCCATCTCATGGGGGCAGATCTGAACAACATTGTGAAATGTCAGAAGCTTACAGATGACCATGTTCAGTTCCTTATCTACCAAATTCTCCGAGGT
MAPK8 (JNK)	NM_002750.2	946-1045	TCTCTGTAGATGAAGCTCTCCAACACCCGTACATCAATGTCTGGTATGATCCTTCTGAAGCAGAAGCTCCACCACCAAAGATCCCTGACAAGCAGTTAGA

MEGF11	NM_032445.2	5096-5195	ACCTCAGTATTTCAGGGCAGCAAGTGTTTGTGAAGCAAGGATGAGCAGCATGAAGATGTCAGATCAGCAAGGAGAAACTTCAACTCAATCCCAGGTTTTC
MEOX1	NM_001040002.1	1711-1810	TCCTCCATGGGATGGATTTAAGCTCTTGCTGTGTGTTCTACAAATGCTGTTATTGTGGGAGGAAATGCTAGGTTTTTGTGTGTGGACTGCCCAGACCTCA
MET	NM_001127500.1	1926-2025	GGCTGACCATATGTGGCTGGGACTTTGGATTTCGGAGGAATAATAAATTTGATTTAAAGAAAACTAGAGTTCTCCTTGGAAATGAGAGCTGCACCTTGAC
MMRN1	NM_007351.2	3271-3370	GCAGACATCCTTTTACTGGTGACAACTGCACTATCAAGCTTGTGGAAGAAAATGCTTTAGCTCCAGATTTTTCCAAAGGATCTTACAGATATGCACCCAT
MS4A6A	NM_152852.2	147-246	GAACAGACTTGCCTAACAACAGGAAACTTGTATGTCTCGAAGTGGCAATTCACACATAAGGCTCCATGACTCCTGAACTCTCACAAATATTAGTTGGCTC
MS4A7	NM_206939.1	717-816	TCACTGTGCTGGAGCTCTTATTAGCTGCATACAGTTCTGTCTTTTGGTGGAAACAGCTCTACTCCAACAACCCTGGGAGTTCATTTTCCTCGACCCAGTC
MYB	NM_001130173.1	184-283	CCGCGCCCGCCGCGCCATGGCCCGAAGACCCCGGCACAGCATATATAGCAGTGACGAGGATGATGAGGACTTTGAGATGTGTGACCATGACTATGATGGG
MYBL1	NM_001080416.3	1031-1130	TTCAACACAAACCTTGTGCAGCTATGGATCATATGCAAACCCAGAATCAGTTTTACATACCTGTTCAGATCCCTGGGTATCAGTATGTGTCACCTGAAGG
NAFTC1	NM_172389.1	1985-2084	CGAATTCTCTGGTGGTTGAGATCCCGCCATTTCGGAATCAGAGGATAACCAGCCCCGTTCACGTCAGTTTCTACGTCTGCAACGGGAAGAGAAAGCGAAG
NAFTC2	NM_012340.3	1816-1915	GACGGACATTGGAAGAAAGAACACGCGGGTGAGACTGGTTTTCCGAGTTCACATCCCAGAGTCCAGTGGCAGAATCGTCTCTTTACAGACTGCATCTAAC
NAFTC3	NM_004555.2	2191-2290	GTCCTTGAAGTTCCTCCATATCATAACCCAGCAGTTACAGCTGCAGTGCAGGTGCACTTTTATCTTTGCAATGGCAAGAGGAAAAAAAGCCAGTCTCAAC
NF-kb 1	NM_003998.2	1676-1775	AGGGTATAGCTTCCCACACTATGGATTTCCTACTTATGGTGGGATTACTTTCCATCCTGGAACTACTAAATCTAATGCTGGGATGAAGCATGGAACCATG
NF-kb 1A	NM_020529.1	946-1045	GGATGAGGAGAGCTATGACACAGAGTCAGAGTTCACGGAGTTCACAGAGGACGAGCTGCCCTATGATGACTGTGTGTTTGGAGGCCAGCGTCTGACGTTA
NF-kb 2	NM_001077493.1	1062-1161	TGTGTTCCGGACACCCCCCTATCACAAGATGAAGATTGAGCGGCCTGTAACAGTGTTTCTGCAACTGAAACGCAAGCGAGGAGGGGACGTGTCTGATTCC
NFKBIZ	NM_001005474.1	2031-2130	ATTTGGTTCCCGATGGCCCTGTGGGAGAACAGATCCGACGTATCCTGAAGGGAAAGTCCATTCAGCAGAGAGCTCCACCGTATTAGCTCCATTAGCTTGG
NKG7	NM_005601.3	633-732	CTGTGGCGGTCCCCGTCCTGGCTATGAAACCTTGTGAGCAGAAGGCAAGAGCGGCAAGATGAGTTTTGAGCGTTGTATTCCAAAGGCCTCATCTGGAGCC
NLRP3	NM_001079821.2	416-515	AGTGGGGTTCAGATAATGCACGTGTTTCGAATCCCACTGTGATATGCCAGGAAGACAGCATTGAAGAGGAGTGGATGGGTTTACTGGAGTACCTTTCGAG
NNMT	NM_006169.2	606-705	GTTCTAAAAGAAGGGCTGAACTGATGGAAGGAATGCTGTTAGCCTGAGACTCAGGAAGACAACTTCTGCAGGGTCACTCCCTGGCTTCTGGAGGAAAGAG
NPHS1	NM_004646.3	4051-4150	CCAGAGTAGAAACAAGGTGCATCCTGGGGTTGGCTTTAGAAACTAAACTTCTCCAAAAGGACAGGGCAGATTGTAAACGTCGTCTCAAAAATGAAATGCT

NPHS2	NM_014625.2	1181-1280	CCTTCCAAACCTGTTGAGCCACTAAATCCTAAAAAGAAAGACTCTCCCATGTTATAGGAA GGATGGGGCATAATGTGACTGTAAAGGGGCCTGCCATAGA
OAZ1	NM_004152.2	314-413	GGTGGGCGAGGGAATAGTCAGAGGGATCACAATCTTTCAGCTAACTTATTCTACTCCGA TGATCGGCTGAATGTAACAGAGGAACTAACGTCCAACGACA
OSMR	NM_003999.2	711-810	CCCTTTGGAATGTGCCACACACTTTGTAAGAATAAAGAGTTTGGTGGACGATGCCAAGTT CCCTGAGCCAAATTTCTGGAGCAACTGGAGTTCCTGGGAG
P2RX7	NM_002562.5	781-880	ACATCACTTGTACCTTCCACAAGACTCAGAATCCACAGTGTCCCATTTTCCGACTAGGAG ACATCTTCCGAGAAACAGGCGATAATTTTTCAGATGTGGC
PALMD	NM_017734.4	261-360	GATCTGTGCATCTGCTCGGAGACGCTCCTGACAAGTCGGGAATTTCTCTATTTCTCCACT GGTGCAAAGAGCGGATTTCTCCCTGCTTCTCTTCTGTCAC
PDGFB	NM_033016.2	1481-1580	GATTCACCTCTTCCTCTGGTTCCTTTCATCTCTCTACCTCCACCCTGCATTTTCCTCTTGT CCTGGCCCTTCAGTCTGCTCCACCAAGGGGCTCTTGAAC
PECAM1	NM_000442.3	1366-1465	ATCTGCACTGCAGGTATTGACAAAGTGGTCAAGAAAAGCAACACAGTCCAGATAGTCGT ATGTGAAATGCTCTCCCAGCCCAGGATTTCTTATGATGCCC
PGM5	XM_024447573.1	264-363	GGCAGCTCGGGCGGTCTGGCAGCGGAGAAGGTGGGTGGGAGAAAACTTTACAGGAAG GCAGAAGCAATCTCTGCCGAGAGAGTCAGCCGAGCGGCCGCGC
PHEX	NM_000444.5	1695-1794	TTAGCAGGCGCTTTCAGTATAGATGGCTGGAATTCTCAAGGGTAATCCAGGGGACCACA ACTTTGCTGCCTCAATGGGACAAATGTGTAAACTTTATTGA
(PI3K) gamma	NM_002649.2	2126-2225	AAAACATACCAATTGTTGGCCAGAAGGGAAGTCTGGGATCAAAGTGCTTTGGATGTTGG GTTAACAATGCAGCTCCTGGACTGCAACTTCTCAGATGAAA
Phospholipase A2 G2A	NM_000300.2	716-815	TTCCCTGGAAACCTTCCACCCAGTGCTGAATTTCCCTCTCTCATACCCTCCCTCCCTACC CTAACCAAGTTCCTTGGCCATGCAGAAAGCATCCCTCACC
PKC (D form)	NM_006254.3	2166-2265	ACCATAAACTGGACTCTGCTGGAAAAGCGGAGGTTGGAGCCACCTTTCAGGCCCAAAGT GAAGTCACCCAGAGACTACAGTAACTTTGACCAGGAGTTCC
PLA1A	NM_015900.2	1251-1350	CAACCGAGTTTGGAAAAAAGACCGGACTACCATTATTGGGAAGTTCTGCACTGCCCTTTT GCCTGTCAATGACAGAGAAAAGATGGTCTGCTTACCTGAA
PLAT	NM_000931.2	1335-1434	CAAGGTTACCAACTACCTAGACTGGATTCGTGACAACATGCGACCGTGACCAGGAACAC CCGACTCCTCAAAAGCAAATGAGATCCCGCCTCTTCTTCTT
PLCG2	NM_002661.2	526-625	GCTTGAAAATCTTACACCAGGAAGCGATGAATGCGTCCACGCCCACCATTATCGAGAGT TGGCTGAGAAAGCAGATATATTCTGTGGATCAAACCAGAAG
POLR2A	NM_000937.2	3776-3875	TTCCAAGAAGCCAAAGACTCCTTCGCTTACTGTCTTCCTGTTGGGCCAGTCCGCTCGAG ATGCTGAGAGAGCCAAGGATATTCTGTGCCGTCTGGAGCAT
PSMB10	NM_002801.2	222-321	ACCATCGCGGGCCTGGTGTTCCAAGACGGGGTCATTCTGGGCGCCGATACGCGAGCCA CTAACGATTCGGTCGTGGCGGACAAGAGCTGCGAGAAGATCC
PSMB9	NM_002800.4	456-555	TCAGGTATATGGAACCCTGGGAGGAATGCTGACTCGACAGCCTTTTGCCATTGGTGGCT CCGGCAGCACCTTTATCTATGGTTATGTGGATGCAGCATAT
PSME2	NM_002818.2	316-415	AGAAGAAAGAAGTCCATAAGTGTGGATTTCTCCCTGGGAATGAGAAAGTCCTGTCCCTG CTTGCCCTGGTTAAGCCAGAAGTCTGGACTCTCAAAGAGAA

PSTPIP1	NM_001321135.1	849-948	GGCCGTCATGGACCGGGTCCAGAAGAGCAAGCTGTCGCTCTACAAGAAGGCCATGGAG TCCAAGAAGACATACGAGCAGAAGTGCCGGGACGCGGACGAC
PTPN7	NM_001199797.1	589-688	CCTGGACATCCCTGGCCACGCCTCCAAGGACCGATACAAGACCATCTTGCCAAATCCCC AGAGCCGTGTCTGTCTAGGCCGGGCACAGAGCCAGGAGGAC
RAMP3	NM_005856.2	834-933	TTTCTATGCTGTTTCTTAGCACAGAATCCAGCCTAGCCTTAGCCGCAGTCTAGGCCCTGC TTGGACTAGGACTCCTTGCTTGACCCCATCTCTGGTTCCT
RAPGEF5	NM_012294.3	3421-3520	GCATTTTGCGTACCTCATACAGGCTCCTTGCCCACACTATGGAATGACAGCAGCCAGTG CAGGGAGGTTAAGTGACATTTAATGAGTGAAGCACTTAGCA
RARRES1	NM_206963.1	607-706	ATAATCATGGACATATTGATCCCTCTCTGAGACTCATCTGGGATTTGGCTTTCCTTGGAA GCTCTTACGTGATGTGGGAAATGACAACACAGGTGTCACA
RARRES3	NM_004585.3	641-740	CTGACCCTCGTGCCCTGTCTCAGGCGTTCTCTAGATCCTTTCCTCTGTTTCCCTCTCTCG CTGGCAAAAGTATGATCTAATTGAAACAAGACTGAAGGAT
RASIP1	NM_017805.2	2469-2568	ACCAGCCAGTGCGAGCTGCACCCTGACCTCGTGTCTCAGACTTTTGGCTACTTGTTCTT CTTCTCCAACGCATCCCTTCTCAACTCGCTGATGGAACGAG
RHOJ	NM_020663.4	231-330	GGGTTTCTTAACTCACACTGAGAGCGGAAAGGGGCAGACCCTTTTCATAACTCCCTCAA GTGTGTGTTACCTTTCTTTACCAGCATGGTAAGCAACAGGA
RNF149	NM_173647.3	687-786	GTTGGCACCCGGCATGTACAGGAGTTCATCAGCGGTCAGTCTGTGGTGTTTGTGGCCAT TGCCTTCATCACCATGATGATTATCTCGTTAGCCTGGCTAA
ROBO4	NM_019055.5	3081-3180	CCTGACTCTCAGATCTCTTCCCAGAGAAGTCAGCTCCACTGTCGTATGCCCAAGGCTGG TGCTTCTCCTGTAGATTACTCCTGAACCGTGTCCCTGAGAC
RORgamma	NM_001001523.1	1351-1450	CTCATCAATGCCCATCGGCCAGGGCTCCAAGAGAAAAGGAAAGTAGAACAGCTGCAGTA CAATCTGGAGCTGGCCTTTCATCATCATCTCTGCAAGACTC
RPS6	NM_001010.2	172-271	GAATGGAAGGGTTATGTGGTCCGAATCAGTGGTGGGAACGACAAACAAGGTTTCCCCAT GAAGCAGGGTGTCTTGACCCATGGCCGTGTCCGCCTGCTAC
RPS6KB1	NM_003161.2	311-410	GAGAAATTTGAAATCTCAGAAACTAGTGTGAACAGAGGGCCAGAAAAAATCAGACCAGA ATGTTTTGAGCTACTTCGGGTACTTGGTAAAGGGGGCTATG
RXRA	NM_002957.4	5051-5150	TACAAATGTAATTTTATCCCTCATGTATACTTGGATATGGCGGGGGGAGGGCTGGGACT GTTTCGTTTCTGCTTCTAGAGATTGAGGTGAAAGCTTCGTC
S1PR1	NM_001400.3	1066-1165	CCTGACGTTCCGCAAGAACATTTCCAAGGCCAGCCGCAGCTCTGAGAAGTCGCTGGCG CTGCTCAAGACCGTAATTATCGTCCTGAGCGTCTTCATCGCC
S1PR5	NM_030760.3	1416-1515	TGCCCTCGTGGAATTGACGTTCTGCTTGGGAACACAGAAAAGAACTCGGTGATGAAATA ATGGAGATGATTCCAGTGACAAACGACAGAGATGGTGATGG
SDHA	NM_004168.1	231-330	TGGAGGGGCAGGCTTGCGAGCTGCATTTGGCCTTTCTGAGGCAGGGTTTAATACAGCAT GTGTTACCAAGCTGTTTCCTACCAGGTCACACACTGTTGCA
SELE	NM_000450.2	1506-1605	AATTCACCTACAAGTCCTCTTGTGCCTTCAGCTGTGAGGAGGGATTTGAATTACATGGAT CAACTCAACTTGAGTGCACATCTCAGGGACAATGGACAGA
SERINC5	NM_001174071.2	527-626	CACAATGGCTTTTGGTTCTTTAAACTTCTGCTGTTGGGGGCCATGTGCTCAGGAGCTTTC TTCATTCCAGATCAGGACACCTTTCTGAACGCCTGGCGCT

SH2D1A	NM_002351.4	496-595	GCTGTATCACGGTTACATTTATACATACCGAGTGTCCCAGACAGAAACAGGTTCTTGGAGTGCTGAGACAGCACCTGGGGTACATAAAAGATATTTCCGG
SH2D1B	NM_053282.4	546-645	GTTGAAGAGATGAGTAACAGTTCTCACTGATGACCCACTTCTGCAGGCATAGGTCCAGAGCACCAAACTCTAGTGGACAATTCAGACTCTCCTGGTTGTG
SHROOM3	NM_020859.3	4705-4804	AGGATGTGCTTTTGGGGCAAGACAGTGGCTTTGGTCTTGTGAAGGATCCATGTTATTTGGCTGGTCCTGGATCTAGGTCACTCAGTTGTTCAGAAAGAGG
SIRPG	NM_001039508.1	132-231	CTTACAGAAGTGGCAGGTGAGGAGGAGCTACAGATGATTCAGCCTGAGAAGCTCCTGTTGGTCACAGTTGGAAAGACAGCCACTCTGCACTGCACTGTGA
SLA	NM_001045556.2	981-1080	GTGTGATTTCTGATGAAGGGGGCTGGTGGAAAGCTATTTCTCTTAGCACTGGTCGAGAGAGTTACATCCCTGGAATATGTGTGGCCAGAGTTTACCATGG
SLAMF8	NM_020125.2	697-796	AGCTGGCGACGGGAGACAACCATGGACTTTGGTATGGAACCACACAGCCTCTTCACAGACGGACAGGTGCTGAGCATTTCCCTGGGACCAGGAGACAGAG
SOD2	NM_000636.2	202-301	TTTGGGGTATCTGGGCTCCAGGCAGAAGCACAGCCTCCCCGACCTGCCCTACGACTACGGCGCCCTGGAACCTCACATCAACGCGCAGATCATGCAGCTG
SOST	NM_025237.2	981-1080	GAGGCAGAAATGGAAGCATTTTCACCGCCCTGGGGTTTTAAGGGAGCGGTGTGGGAGTGGGAAAGTCCAGGGACTGGTTAAGAAAGTTGGATAAGATTCC
SOX7	NM_031439.2	2636-2735	CTGTGAGAATTTGTCTTCCTCACCAGCCAGGTCCTCAGGCAAAGTCCTCAGCCAGTGCTTTAGAGCAACTTCCCGCAAATCAGAAACTCACTGTGATTCC
SP140	NM_001005176.2	246-345	GGTTCTTCAGAGAAAACAAGGTGGAGATTGCAAGTGCAATAACAAGGCCATTTCCTTTCCTTATGGGCCTCCGAGACCGCTCCTTCATCTCCGAGCAGAT
SPLI	NM_003064.2	331-430	TTTCTGTGAGATGGATGGCCAGTGCAAGCGTGACTTGAAGTGTTGCATGGGCATGTGTGGGAAATCCTGCGTTTCCCCTGTGAAAGCTTGATTCCTGCCA
SPRY4	NM_030964.3	1901-2000	CTCCCTTCCAACTGCATCAACTAACTCTCGGGGGTGTTCTGCTCACCACACCGTCCTTCGGTTCTTACTGAGTCACAGACTCGCCTGCCCACTACGTGTC
ST5	NM_005418.3	2619-2718	TTTTGCTTCCCTGATGCCAAGGACTGGCTTCCTGTGTCAGAGTATAGCAGTGAGACCTTTTCTTTCATGCTGACTGGGGAAGATGGCAGCAGACGCTTTG
ST8SIA4	NM_005668.4	696-795	ATCTACATAGCCTCCTACCTGAAGTTTCACCAATGAAGAATCGCAGGTTTAAGACCTGTGCAGTTGTTGGAAATTCTGGCATTCTGTTAGACAGTGAATG
TAP1	NM_000593.5	2076-2175	GTGGCTGCAGTGGGACAAGAGCCACAGGTATTTGGAAGAAGTCTTCAAGAAAATATTGCCTATGGCCTGACCCAGAAGCCAACTATGGAGGAAATCACAG
TBX21	NM_013351.1	891-990	ACACAGGAGCGCACTGGATGCGCCAGGAAGTTTCATTTGGGAAACTAAAGCTCACAAACAACAAGGGGGCGTCCAACAATGTGACCCAGATGATTGTGCT
TEK	NM_000459.3	1895-1994	CCCGTTAATCACTATGAGGCTTGGCAACATATTCAAGTGACAAATGAGATTGTTACACTCAACTATTTGGAACCTCGGACAGAATATGAACTCTGTGTGC
TGIF1	NM_170695.2	1261-1360	CCGGGATCAGTTTTGGCTCGTCCATCAGTGATCTGCCATACCACTGTGACTGCATTGAAAGATGTCCCTTTCTCTCTCTGCCAGTCGGTCGGTGTGGGAC
THBD	NM_000361.2	1247-1346	GTGGAGCCCGTGGACCCGTGCTTCAGAGCCAACTGCGAGTACCAGTGCCAGCCCCTGAACCAAACTAGCTACCTCTGCGTCTGCGCCGAGGGCTTCGCGC

TIGIT	NM_173799.2	1969-2068	TGGATCTTAGAAGACTTTTATCCTTCCACCATCTCTCTCAGAGGAATGAGCGGGGAGGTTGGATTTACTGGTGACTGATTTTCTTTCATGGGCCAAGGAA
TM4SF1	NM_014220.2	96-195	AATCGCAGTATTTAAGAGGTAGCAGGAATGGGCTGAGAGTGGTGTTTGCTTTCTCCACCAGAAGGGCACACTTTCATCTAATTTGGGGTATCACTGAGCT
TM4SF18	NM_138786.3	2414-2513	CCCCAGAGTCATGGGCAGCCTATTGTCTTGCTAATGGCCTCTGACTTAGCCTGGAGTAAAACACTGTCCAAACAAGACTGGTGGTTATTCAAGCATAAGA
TNF	NM_000594.2	1011-1110	AGCAACAAGACCACCACTTCGAAACCTGGGATTCAGGAATGTGTGGCCTGCACAGTGAAGTGCTGGCAACCACTAAGAATTCAAACTGGGGCCTCCAGAA
TNFSF8	NM_001244.3	400-499	CAGCTATTTCTATTTGACCACAGCCACTCTGGCTCTGTGCCTTGTCTTCACGGTGGCCACTATTATGGTGTTGGTCGTTCAGAGGACGGACTCCATTCCC
TOX2	NM_001098798.1	785-884	GAAGCCTGTGTCGGCCTACGCACTCTTCTTCAGAGACACTCAGGCCGCCATCAAGGGTCAGAACCCCAGTGCCACTTTCGGTGACGTGTCCAAAATCGTG
TRD	M21624.1	786-885	AGCAACCTTCAAAGAGCTGCCATAAACCCAAAGCCATAGTTCATACCGAGAAGGTGAACATGATGTCCCTCACAGTGCTTGGGCTACGAATGCTGTTTGC
TRDV3	ENST00000535880.1	288-387	GGATAACAGCAGATCAGAAGGTGCAGATTTTACTCAAGGACGGTTTTCTGTGAAACACATTCTGACCCAGAAAGCCTTTCACTTGGTGATCTCTCCAGTA
TRIB1	NM_025195.2	3461-3560	CTCGACTGTTGTATCTGTGATACATTATCCGACTAAGGACTCTGGGCTGGCAGGGCCTTCTGCCGGGAAAGCTAGAAACACTAGGTTCTTCCTGTACATA
UBB	NM_018955.2	796-895	CACCTGGTCCTGCGCCTGAGGGGTGGCTGTTAATTCTTCAGTCATGGCATTCGCAGTGCCCAGTGATGGCATTACTCTGCACTATAGCCATTTGCCCCAA
VAV1	NM_005428.2	765-864	CCCTGCAACGGTTCCTGAAACCTCAAGACATTGAGATCATCTTTATCAACATTGAGGACCTGCTTCGTGTTCATACTCACTTCCTAAAGGAGATGAAGGA
VAV2	NM_001134398.1	1616-1715	CACAGTTTCCAGATGTACACGTTTGACAAGACCACCAACTGCAAAGCCTGCAAAATGTTCCTCAGGGGCACCTTCTACCAGGGATACATGTGTACCAAGT
VAV3	NM_001079874.1	353-452	GCAGTCAAGCCTTGCCCATGTGTGCCCAAACCAGTAGATTATTCTTGCCAACCCTGGTATGCTGGAGCAATGGAAAGATTGCAAGCAGAGACCGAACTTA
VCAN	NM_004385.3	9916-10015	GCAGGGTGCCCATCTCACAAGCATCCTGTCTCACGAAGAACAAATGTTTGTTAATCGTGTGGGCCATGATTATCAGTGGATAGGCCTCAATGACAAGATG
VMP1	NM_030938.3	931-1030	GATGATGAAGAGTATCAGGAATTTGAAGAGATGCTGGAACATGCAGAGTCTGCACAAGACTTTGCCTCCCGGGCCAAACTGGCAGTTCAAAAACTAGTAC
VWF	NM_000552.3	8116-8215	CACCTGCAACCCCTGCCCCCTGGGTTACAAGGAAGAAAATAACACAGGTGAATGTTGTGGGAGATGTTTGCCTACGGCTTGCACCATTCAGCTAAGAGGA
WNT9A	NM_003395.1	556-655	TCCTGGGCAGACGGTCAAGCAAGGATCTGCGAGCCCGTGTGGACTTCCACAACAACCTCGTGGGTGTGAAGGTGATCAAGGCTGGGGTGGAGACCACCTG
